# Planar Chiral Nanoantenna for Excitation‐Chirality‐Controlled Hot Spot Modulation and Emitter‐Coupled Circularly Polarized Emission

**DOI:** 10.1002/nap2.70199

**Published:** 2026-07-14

**Authors:** Abhik Chakraborty, Xiaofei Wu, Ankit Kumar Singh, Fabian Scheidler, Min Jiang, Jürgen Popp, Bert Hecht, Jer‐Shing Huang

**Affiliations:** ^1^ Institute of Physical Chemistry Friedrich Schiller University Jena Jena Germany; ^2^ Abbe Center of Photonics Friedrich Schiller University Jena Jena Germany; ^3^ Leibniz Institute of Photonic Technology Jena Germany; ^4^ Experimental Physics 5 University of Würzburg Würzburg Germany; ^5^ ARC Centre of Excellence for Transformative Meta‐Optical Systems, Department of Electronic Materials Engineering Research School of Physics The Australian National University Canberra Australia; ^6^ Research Center for Applied Sciences Academia Sinica Taipei Taiwan; ^7^ Department of Electrophysics National Yang Ming Chiao Tung University Hsinchu Taiwan

**Keywords:** chirality, circularly polarized emission, dissymmetry factor, plasmonic hot spot, polarimetry

## Abstract

A planar chiral plasmonic nanoantenna exhibiting an excitation‐chirality‐dependent hot spot in a nanogap is numerically investigated. Additionally, the underlying design principles are examined, providing a broadly applicable framework for engineering chiral nanoantennas through controlled geometrical or modal asymmetry. The hot spot can be turned on and off by changing the handedness of the exciting circularly polarized light (CPL). This effect stems from the rationally designed interference of plasmonic modes excited by the linearly polarized orthogonal components of CPL. The hot spot exhibits maximal near‐field dissymmetry factor (≈ −2) at a wavelength of 842 nm. The intensity at the hot spot can also be continuously modulated by varying the excitation ellipticity and handedness, approaching a modulation depth of 100%. These attributes enable chirality‐ and ellipticity‐dependent switching and dynamic modulation of the plasmonic near field. Moreover, placing an achiral quantum emitter in the nanogap generates almost perfectly circularly polarized emission, offering a simple yet effective avenue to realize nanoscale circularly polarized single‐photon sources.

## Graphical abstract

A planar chiral plasmonic nanoantenna is shown to translate the changing handedness of circularly polarized excitation into deterministic switching of a nanoscale hot spot with maximal dissymmetry and complete modulation depth. Coupling an achiral quantum emitter to the nanogap simultaneously enables circularly polarized emission of near‐maximal purity, linking chiral near‐field control with spin‐selective far‐field engineering.

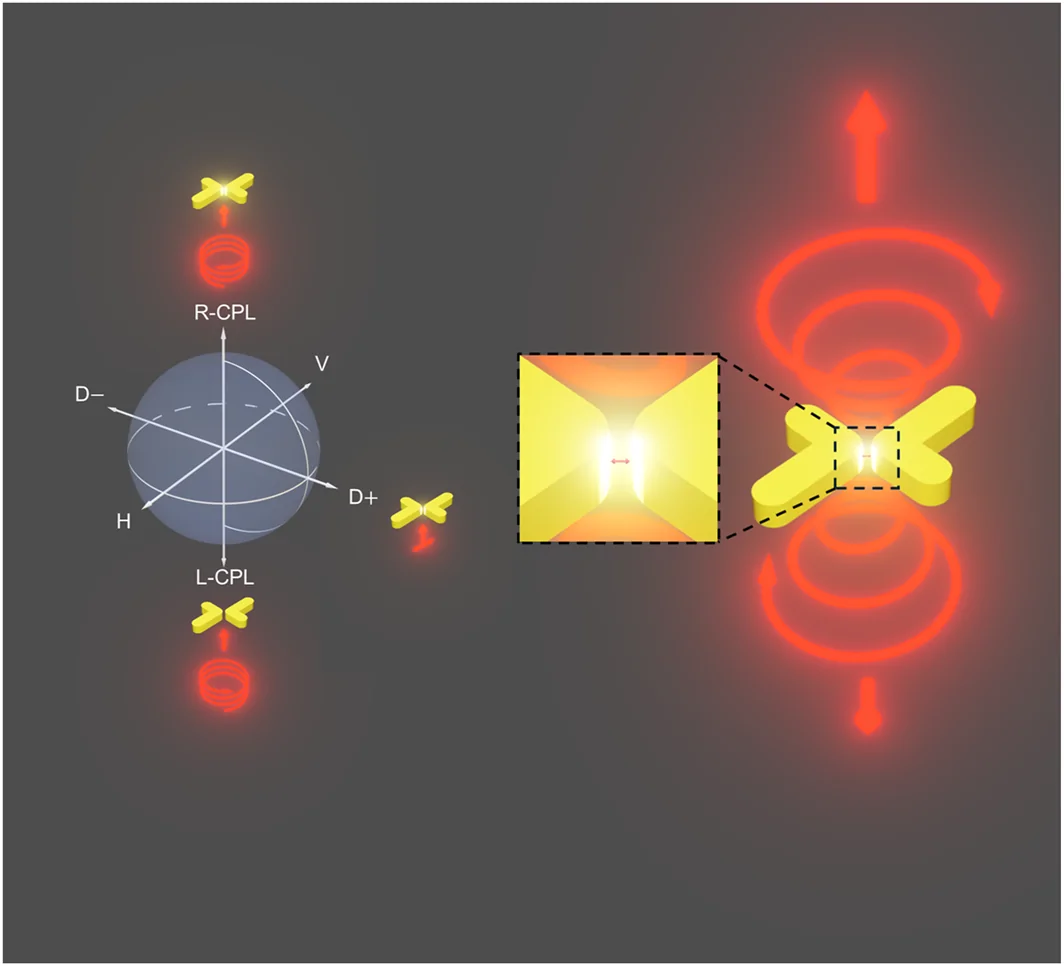

## 1 Introduction

General limitations to the efficiency of light–matter interaction at the mesoscopic to microscopic regime are the diffraction limit [1] and the size mismatch between the wavelength of light and an individual emitter [2]. Plasmonic nanostructures overcome these limitations and enhance weak light–matter interaction mainly through two complementary mechanisms. First, plasmonic hot spots enforce the concentration of electromagnetic fields into deep sub‐wavelength volumes, thus attaining sub‐diffraction spatial resolution. This also leads to highly increased interaction probability through enhanced near‐field intensity, enhanced local density of optical states, and a broadband near‐field momentum distribution. Second, plasmonic nanostructures host electromagnetic modes that facilitate antenna‐like radiative and resonator‐like nonradiative behaviors, which can also be strategically engineered or selected to tailor coupling efficiencies and emission rates. In applications involving naturally forbidden light–matter interactions, such as intraband transitions typical of photoluminescence in gold [3] or brightening of dark excitons in 2D materials [4], these complementary mechanisms enhance the coupling efficiency and radiative emission rate. In the case of nonlinear optics [5, 6], where phase‐matching constraints, higher‐order corrections to material polarizability, and small scattering cross‐sections are crucial features, plasmonic nanostructures enhance the inherently weak interaction and resultant optical signals in a similar manner. The applicability of plasmonic nanostructures acting as rich playgrounds for light–matter interaction extends to domains as diverse as nanometrology [7], photocatalysis [8], color generation [9], lasing [10], quantum optics [11, 12], and more.

Coherent interaction or interference of spatially overlapping plasmonic modes in rationally designed nanostructures can be employed to optimally engineer vital attributes of the electromagnetic field, such as amplitude, phase, polarization, radiative directionality, pulse duration, group velocity, etc. One particularly compelling direction of research where interference of plasmonic modes becomes pivotal is the field of chiral nanoplasmonics [13]. Chirality is a geometric property characteristic of an object that cannot be superimposed on its mirror image. Chiral objects upon interaction with circularly polarized light (CPL) respond differently depending on the agreement or disagreement in handedness between the chiral object and CPL [14]. Much of the recent progress in chiral nanoplasmonics has centered on handedness‐selective optical chirality enhancement in the near field for enantioselective sensing [15]. Other topics of considerable interest related to handedness‐dependent response include differential scattering [16] and directionality switching [17]. Beyond nanoplasmonics, several dielectric alternatives have also proven to be promising platforms for chiroptical nanophotonics [18, 19, 20]. In contrast to the aforementioned topics of chiroptical interest, this work shifts the focus from passive chiroptical sensing and dissymmetric scattering to deterministic, excitation‐chirality‐controlled near‐field intensity switching in the nanogap of a planar chiral nanoantenna for active manipulation of light–matter interactions. This is achieved via the excitation‐chirality‐dependent modal interference, where one excitation handedness concentrates the near field into the deeply sub‐diffraction‐limited nanogap whereas the other excitation handedness redistributes the near field over the larger nanoantenna volume. By reciprocity, the same structured near field can also convert the emission of a single achiral quantum emitter into highly circularly polarized emission in the far field. The combination of deep sub‐diffraction confinement of the electric near field, excitation‐chirality‐controlled switching of the confined mode, and the reciprocal generation of highly circularly polarized emission from a single achiral quantum emitter firmly grounds the proposed planar chiral nanoantenna as an effective platform for chiral control of light at the nanoscale.

The first part of our work studies the on/off switching of a localized hot spot in the nanogap by changing the handedness of the illuminating CPL. The chiral sensitivity arises from the interference of plasmonic modes excited by the linearly polarized orthogonal components of CPL, allowing for the hot spot to be selectively activated or deactivated by simply switching the handedness of CPL. Notably, the nanoantenna demonstrates the value of the near‐field dissymmetry factor (ɡ) to be approximately −2 in the nanogap. It is important to note here that the maximum value of ɡ is ± 2. Moreover, by varying the excitation ellipticity and handedness, we show that the hot spot intensity can be continuously modulated with a modulation depth approaching 100%. The nanoantenna also exhibits a large absolute contrast in near‐field intensity enhancement (ΔIE) in the nanogap, ensuring that the observed dissymmetry arises not only from relative contrast but also from a substantial difference in the local electric field intensity. The ability to turn on and off a hot spot by changing the handedness of CPL or to tune the near‐field hot spot intensity as a function of optical ellipticity and handedness promises tunable near‐field control of light–matter interactions, with applications such as polarimetry [21], polarization‐sensitive circuitry [22, 23, 24], light‐driven nanomotors [25, 26], etc. In particular, emitter‐coupled or nanostructure‐induced coherent nonlinear processes [27, 28, 29, 30, 31, 32] can act as instantaneous probes of the excitation‐chirality‐controlled hot spot. In the second part of this work, we demonstrate that when an achiral dipolar quantum emitter is in the nanogap, its emission polarization can possess a near‐maximal average degree of circular polarization (〈DoCP〉) with its value reaching −0.99 over a large angular range. The maximum possible value of 〈DoCP〉 is ± 1. This allows for precise engineering of the handedness and polarization purity of emitted light, providing a deterministic route toward spin‐polarized single‐photon sources [33, 34], polarization‐engineering plasmonic pixels [35], and on‐chip optical information processing [36, 37]. Attributes such as maximal ɡ, complete modulation depth, very high ΔIE, and near‐maximal 〈DoCP〉 establish a diverse and distinctive set of functionalities within a single chiral nanoplasmonic platform.

## 2 Geometry of the Planar Chiral Nanoantenna and Excitation‐Chirality‐Controlled Hot Spot

The design criteria of the planar chiral nanoantenna demand two specific attributes: a single plasmonic hot spot and planar chiral symmetry breaking. The latter arises intrinsically in structures that lack mirror symmetry along both in‐plane axes, ensuring distinct optical responses to differently handed CPL excitation. As depicted in Figure 1a, to achieve a single and well‐defined hot spot, our nanoantenna consists of two V‐shaped elements positioned with their apexes facing each other, forming a nanogap of width W. W is 10 nm. The two nanorods constituting each V‐shaped element overlap at the aforementioned apex. This configuration ensures strong and localized field confinement in the nanogap, a prerequisite for achieving controlled near‐field enhancement and modulation. However, simply using symmetric V‐shaped elements would result in an achiral structure. To introduce chirality into the system, we start off by designing each V‐shaped element with two coplanar nanorods of unequal lengths: a short rod of length L_1_ and a long rod of length L_2_. Therefore, each V‐shaped element is intrinsically asymmetric. Furthermore, whereas both V‐shaped elements use the same pair of nanorods with lengths L_1_ and L_2_, the relative positioning of the nanorods is inverted in one element in relation to the other. In the design central to this work and exhibited in Figure 1a, the left V‐shaped element's nanorod with length L_1_ extends into the upper‐left quadrant and that with length L_2_ into the lower‐left quadrant from the top‐view perspective. Conversely, for the right V‐shaped element, the nanorod with length L_2_ is at the upper‐right quadrant and that with length L_1_ is at the lower‐right quadrant. The resultant geometry composed of two V‐shaped elements collectively breaks mirror symmetry, and therefore, manifests planar chirality. In our design, L_1_ is 92 nm and L_2_ is 112 nm. Each nanorod has a width (B) of 40 nm and a thickness (T) of 30 nm. The angle (*α*) between the two nanorods in each asymmetric V‐shaped element is 90°. The chosen geometrical parameters satisfy the demonstrated fabrication capabilities of state‐of‐the‐art ion beam milling techniques [25, 26, 29, 32]. The inset of Figure 1a exhibits the top‐view perspective of the nanoantenna, where x and y are the in‐plane polarization states in the near field oriented along and perpendicular to the nanogap axis, respectively. Similarly, p and s are the excitation's linear polarization states oriented along and perpendicular to the nanogap axis, respectively. In this work, the handedness of CPL excitation is defined from the perspective of the receiver. Left‐handed CPL (L‐CPL) corresponds to a phase difference of $\phi_{s}-\phi_{p}=+90{}^{\circ}$, whereas right‐handed CPL (R‐CPL) corresponds to $\phi_{s}-\phi_{p}=-90{}^{\circ}$.

**FIGURE 1:**
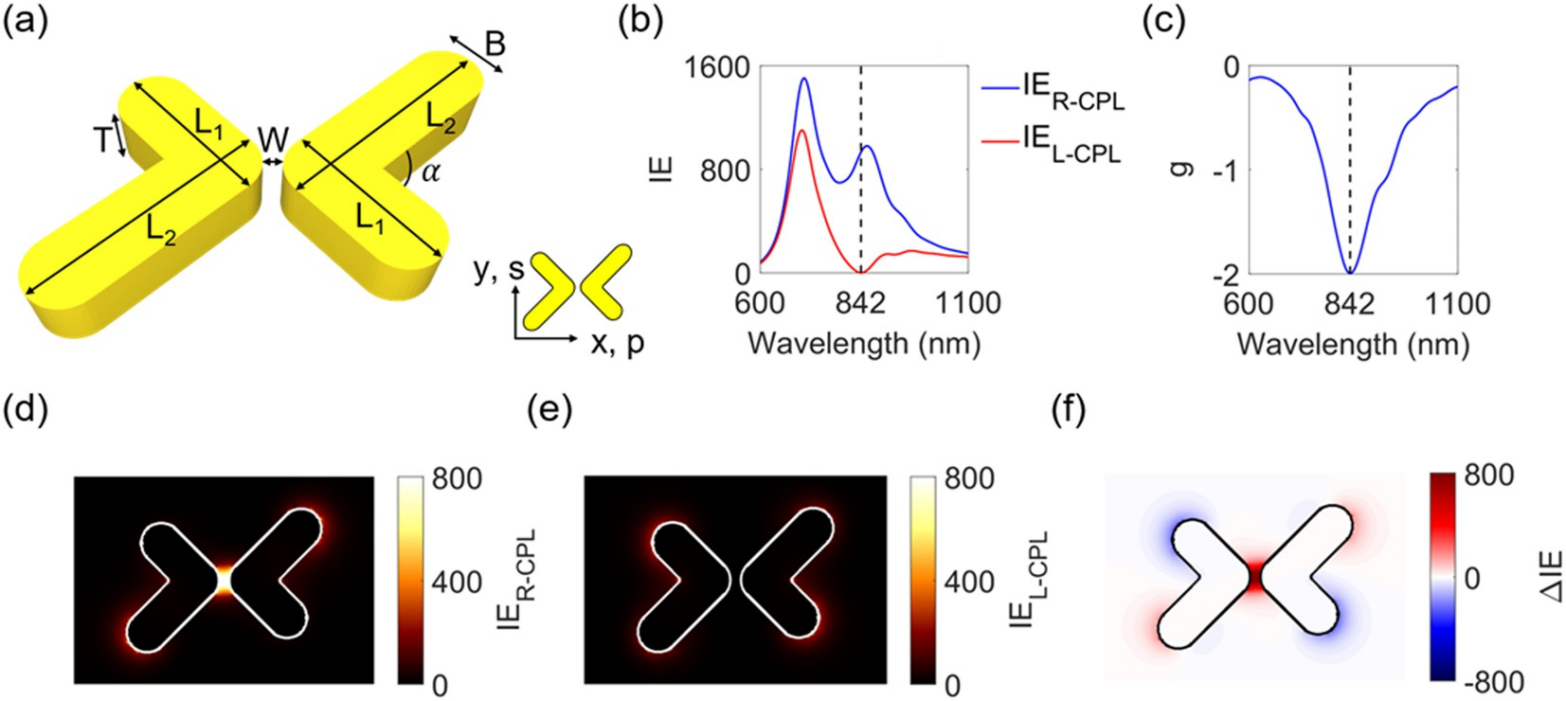
(a) Scheme of a planar chiral nanoantenna consisting of two asymmetric V‐shaped elements. Each element consists of two nanorods of unequal lengths which overlap at the apex of the V. The nanorods' lengths L_1_, L_2_, width B, and thickness T are 92 nm, 112 nm, 40 nm, and 30 nm, respectively. The angle between nanorods *α* and nanogap width W are 90° and 10 nm, respectively. The inset presents a top‐view perspective of the nanoantenna. It elucidates that x and y are the in‐plane near‐field polarization states along and perpendicular to the nanogap axis, respectively, whereas p and s are the excitation polarization states along and perpendicular to the nanogap axis, respectively. (b) Intensity enhancement (IE) spectra at the center of the plasmonic nanogap under right‐handed circularly polarized light (R‐CPL) and left‐handed circularly polarized light (L‐CPL) excitation. The maximum contrast between IE_R‐CPL_ (blue) and IE_L‐CPL_ (red) is observed at a wavelength of 842 nm (vertical dashed line), where IE_R‐CPL_ is 931.68 and IE_L‐CPL_ is 0.98. (c) ɡ spectrum exhibits maximal value of ≈ −2 at 842 nm (vertical dashed line). (d, e) Spatial distribution of IE_R‐CPL_ and IE_L‐CPL_ at the wavelength of 842 nm calculated at a plane positioned mid‐height of the nanoantenna. (f) Spatial distribution of ΔIE, that is, difference between IE_R‐CPL_ and IE_L‐CPL_ at the wavelength of 842 nm calculated at the same plane. The planar chiral nanoantenna dimensions considered in the simulations are identical to the dimensions specified in Panel (a).

Finite‐Difference Time‐Domain (FDTD) simulations are conducted to simulate the response of the nanoantenna depicted in Figure 1a situated on a glass substrate. To investigate the excitation‐chirality‐selective near‐field response of the plasmonic nanoantenna, we simulate the intensity enhancement (IE) spectra at the geometric center of the nanogap under R‐CPL and L‐CPL illumination. The aforementioned IE spectra under R‐CPL and L‐CPL illumination are defined as IE_R‐CPL_ (blue in Figure 1b) and IE_L‐CPL_ (red in Figure 1b), respectively. A significant difference between IE_R‐CPL_ and IE_L‐CPL_ spectra is observed in Figure 1b, with the maximum contrast being observed at a wavelength of 842 nm, where IE_R‐CPL_ is 931.68 and IE_L‐CPL_ is 0.98. This disparity in near‐field intensity enhancement is quantified using ɡ as follows,

(1) $$\begin{array}{c} ɡ=\frac{{\text{IE}}_{L‐\text{CPL}}-{\mathrm{IE}}_{R‐\text{CPL}}}{{\text{IE}}_{L‐\text{CPL}}+{\text{IE}}_{R‐\text{CPL}}}\times 2 \end{array}.$$

Figure 1c shows that ɡ at 842 nm is approximately −2, which is the maximum possible value. Spatial IE maps at 842 nm calculated in a plane situated at mid‐height of the nanoantenna reveal a strong R‐CPL‐induced hot spot with IE_R‐CPL_ exceeding 800 within the nanogap (Figure 1d), in contrast to negligible IE_L‐CPL_ (Figure 1e). This is further confirmed in Figure 1f by the differential intensity enhancement map ΔIE (IE_R‐CPL_ – IE_L‐CPL_) calculated at the same plane and wavelength. It can be seen that ΔIE is confined to the nanogap with its magnitude exceeding 800. This means that the designed nanoantenna is not only capable of yielding a single hot spot with maximal relative contrast in intensity (ɡ ≈ −2) but also provides a very high absolute contrast (ΔIE) of 930.7. These results collectively highlight both pronounced IE in the nanogap and a switchable single plasmonic hot spot with very high on/off ratio controlled by the handedness of CPL. A comprehensive analysis of the dissymmetric near‐field response in the Supporting Information demonstrates robustness of the excitation‐chirality‐dependent switching mechanism against structural variations in W (Supporting Information S1: Figure S1) as well as in L_1_ and L_2_ (Supporting Information S1: Figures S2 and S3). The stability of the dissymmetric near‐field response is further tested in the Supporting Information against variations in illumination parameters such as angle of incidence (Supporting Information S1: Figure S4) and illumination direction (Supporting Information S1: Figure S5). Collectively, the discussed analyses confirm substantial tolerance to possible fabrication‐related deviations and reasonable changes in experimental conditions.

## 3 Origin of the Near‐Field Chiroptical Response

To achieve excitation‐chirality‐dependent switching of the plasmonic hot spot in the nanogap, the nanoantenna design must satisfy the following conditions:

- Co‐localization of the electric near field: The linearly polarized constituents of CPL, that is, p‐ and s‐polarized components, must excite plasmonic modes that produce spatially overlapping electric near fields in the nanogap.
- Co‐polarization of the electric near field: Despite the orthogonality of the excitation polarization states, the co‐localized electric near fields must have the same polarization.
- Equal amplitude contribution: The amplitudes of the co‐localized and co‐polarized electric near fields generated by p‐ and s‐polarized excitations must be approximately equal in the nanogap.
- Phase difference of $\pm (2m+1)\times \;90{}^{\circ},m\in N_{0}$: The co‐localized and co‐polarized electric near fields of approximately equal amplitude generated by s‐ and p‐polarized excitation must maintain a phase difference of approximately an odd multiple of 90°, that is, $\pm \;\left(2m+1\right)\times 90{}^{\circ},m\in N_{0}$. When driven under CPL excitation, an additional phase difference of ± 90° is contributed by the handedness‐dependent phase difference between the s‐ and p‐polarized components of CPL. Therefore, the net phase difference can be written as $\pm \left(\left(2m+1\right)\times 90{}^{\circ}\pm 90{}^{\circ}\right),m\in N_{0}$. Depending on the interplay of two factors, namely the parity of m and the agreement or disagreement between the signs of the modal phase difference in the nanogap and the excitation‐chirality‐dependent phase difference of CPL, the net phase difference equals either an even multiple of $180{}^{\circ}\;\left(\pm 2n\times 180{}^{\circ},n\in N_{0}\right)$ or an odd multiple of $180{}^{\circ}\;\left(\pm \left(2n+1\right)\times 180{}^{\circ},n\in N_{0}\right)$.

Therefore, these conditions collectively enforce a binary switching behavior of either constructive interference (hot spot “on”) with a net phase difference of an even multiple of 180°, or destructive interference (hot spot “off”) with a net phase difference of an odd multiple of 180° [21]. We compare three nanoantenna geometries considering the design criteria discussed above: fully symmetric geometry, partially symmetric geometry, and planar chiral geometry. The following section delves into each nanoantenna geometry to illustrate the role of structural asymmetry and consequent modal interference (see Supporting Information S1: Figure S6 for additional information) in endowing chiroptical attributes in the optimal design.

Previously, it was mentioned that the designed nanoantenna with planar chirality has two asymmetric V‐shaped elements where each element has two rods of length L_1_ (92 nm) and L_2_ (112 nm) with inverted orientational configuration in relation to each other. In contrast to that, the fully symmetric nanoantenna shown in the inset of Figure 2a has two symmetric V‐shaped elements, where all the rods have the identical length of $\frac{L_{1}+L_{2}}{2}$ (102 nm). Therefore, the structure has complete in‐plane mirror symmetry. The mirror planes are indicated by the two orthogonally oriented lines in the inset. On the other hand, the partially symmetric nanoantenna depicted in the inset of Figure 2b consists of two asymmetric V‐shaped elements, each with rod lengths L_1_ and L_2_. However, unlike the planar chiral design, the two V‐shaped elements are not inverted relative to each other. This implies that the structure preserves mirror symmetry along one in‐plane axis. The vertical line in the inset of Figure 2b is the axis across which mirror symmetry is preserved for the partially symmetric nanoantenna. Figure 2a,b make it clear that p‐polarized excitation (blue) generates x‐polarized nanogap‐confined amplitude enhancement (AE) in both cases. Both hot spots are predominantly x‐polarized due to the geometry‐imposed confinement along the same axis. However, Figure 2a,b also show that s‐polarized excitation does not generate x‐polarized AE in the nanogap due to symmetry‐induced restrictions in both cases (see Supporting Information S1: Figure S6 for additional information). Therefore, the design criteria are not met which, in turn, makes both geometrical configurations unsuitable for excitation‐chirality‐dependent near‐field response.

**FIGURE 2:**
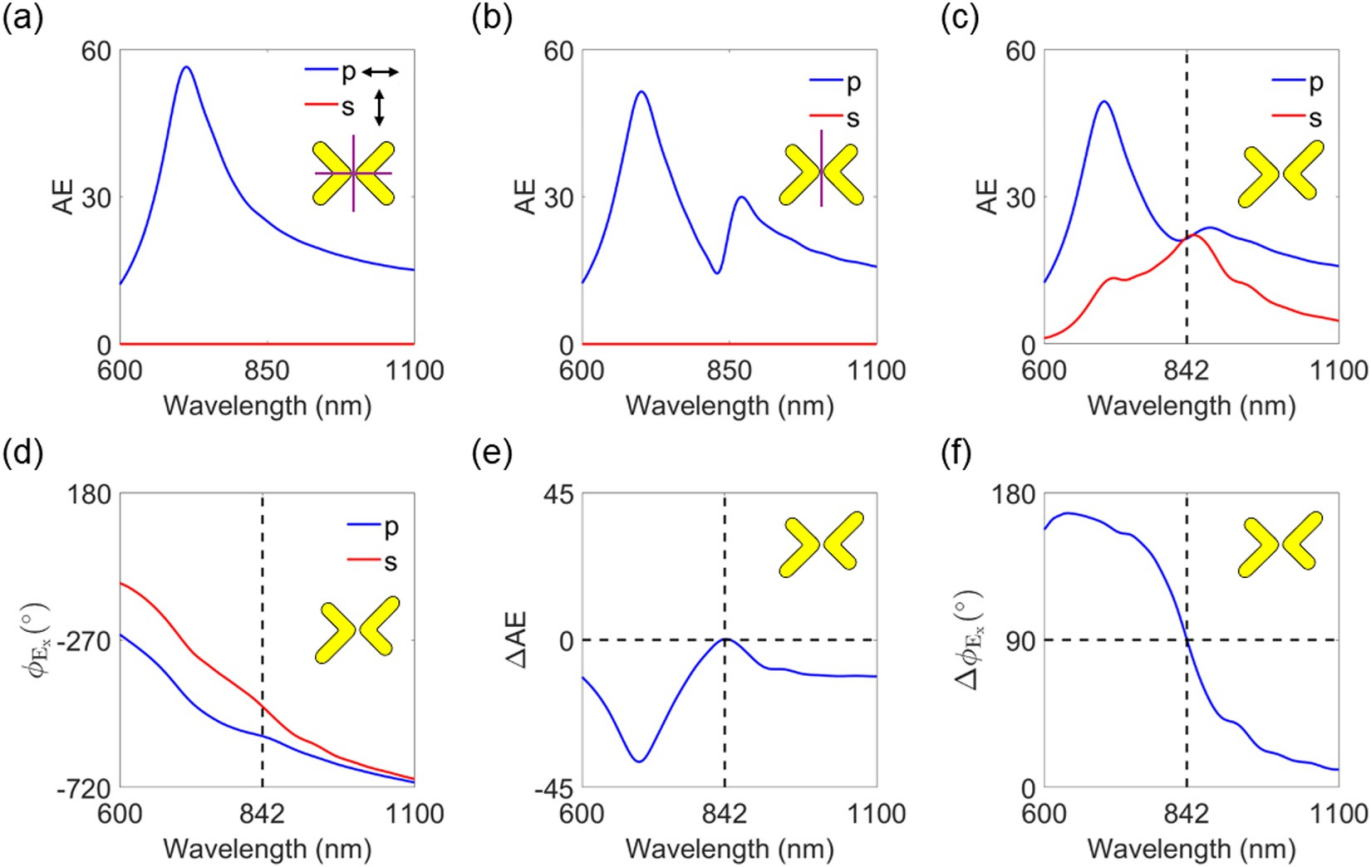
Simulated amplitude enhancement (AE) of the x‐polarized electric near field with respect to wavelength recorded at the center of the nanogap of (a) a fully symmetric, (b) a partially symmetric, and (c) a planar chiral nanoantenna illuminated with p‐ (blue) and s‐polarized (red) excitation. The insets show the nanoantenna geometries in all three cases, where the lines indicate the mirror planes in (a) and (b). (a, b) x‐polarized AE spectra in the nanogaps of the fully symmetric and partially symmetric nanoantennas under p‐polarized illumination, but no such response is observed under s‐polarized illumination. (c) x‐polarized AE spectra in the nanogap of the planar chiral nanoantenna under both p‐ and s‐polarized illumination. (d) The phase spectra ($\phi_{E_{x}}$) of the co‐polarized electric near field recorded in the nanogap of the planar chiral nanoantenna under p‐ and s‐polarized illumination. At 842 nm (vertical dashed line), the co‐polarized electric near fields co‐localized in the nanogap of the planar chiral nanoantenna exhibit (e) amplitude difference ΔAE approaching 0 (horizontal dashed line) and (f) phase difference ${\Delta \phi }_{E_{x}}$ around 90° (horizontal dashed line).

The planar chiral configuration depicted in the inset of Figure 2c lacks mirror symmetry across both in‐plane axes. As shown in Figure 2c, the in‐plane asymmetry of the planar chiral nanoantenna ensures that both p‐ and s‐polarized excitations generate co‐polarized (x‐polarized) hot spots co‐localized in the nanogap. Very importantly, Figure 2c shows that the x‐polarized fundamental mode recorded at the center of the nanogap under p‐polarized and s‐polarized excitation have approximately equal value at and around the wavelength of 842 nm. Since the primary design criteria of co‐localization, co‐polarization, and equal amplitude contribution are satisfied for the fundamental mode of the planar chiral nanoantenna, the next step is to test the criterion for inducing a phase difference of $\pm \left(2m+1\right)\times \;90{}^{\circ},m\in N_{0}$ between the fundamental mode excited by s‐polarized source and the same excited by p‐polarized source at 842 nm. Figure 2d exhibits the phase $\phi_{E_{x}}$ (°) of the co‐localized and co‐polarized fields excited by the orthogonally polarized sources recorded at the center of the nanogap.

Figure 2e confirms that at the wavelength of 842 nm, the difference in AE $\left(\Delta \text{AE}={\text{AE}}_{s}-{\text{AE}}_{p}\right)$ of the two co‐polarized fundamental modes co‐localized in the nanogap under s‐polarized and p‐polarized excitation is approximately 0. Furthermore, Figure 2f shows that at 842 nm, the phase difference $\left(\Delta \phi_{E_{x}}=\phi_{{E_{x}}_{s}}-\phi_{{E_{x}}_{p}}\right)$ between the two nanogap modes is approximately 90°. The additional phase difference of $\phi_{s}-\phi_{p}=-90{}^{\circ}$ associated with R‐CPL excitation leads to a net phase difference of approximately 0°, leading to constructive interference and hot spot in the nanogap. Under L‐CPL excitation ($\phi_{s}-\phi_{p}=+90{}^{\circ}$), the net phase difference is approximately 180°, implying destructive interference in the nanogap. The conclusions explain the results exhibited in Figure 1b. Analysis of the identical extinction response of the planar chiral nanoantenna under oppositely handed excitation (Supporting Information S1: Figure S7) further supports the phase‐driven origin of the near‐field switching mechanism. Therefore, from Figure 2e,f, it becomes clear that the planar chiral nanoantenna, owing to its in‐plane structural asymmetry, satisfies the complete set of design criteria, thus making sure that CPL of opposite handedness leads to constructive or destructive interference in the nanogap which, in turn, switches the hot spot on or off.

## 4 Modulation of Hot Spot Intensity and ɡ With Excitation Ellipticity and Handedness

Figure 3 presents a comprehensive analysis of the chiral near‐field response enabled by the planar chiral nanoantenna. In Figure 3a, a Poincaré sphere representation illustrates the modulation of IE at the center of the nanogap as a function of the excitation polarization state at the wavelength of 842 nm. A continuous trajectory from R‐CPL to L‐CPL incidence passing through the D+ linear polarization point (oriented at 45° to p) reveals a tunable response governed solely by the phase difference between the linearly polarized orthogonal components. IE reaches a value of 931.68 under R‐CPL excitation and diminishes monotonically to 0.98 under L‐CPL, demonstrating precise control of the hot spot intensity via incident polarization's handedness and ellipticity. The continuous tunability is remarkable as it achieves a modulation depth $\left(\left|\frac{I_{L‐\text{CPL}}-I_{R‐\text{CPL}}}{I_{R‐\text{CPL}}}\right|\times 100\right)$ of approximately 100%. Figure 3b quantifies this behavior further by plotting ɡ with respect to wavelength at various excitation ellipticities determined by the absolute phase difference between the linearly polarized orthogonal components of the excitation, that is, $\left|\phi_{s}-\phi_{p}\right|$. The global maximum ɡ of approximately −2 occurs at 842 nm under CPL $\left(\left|\phi_{s}-\phi_{p}\right|=90{}^{\circ}\right)$ while reducing phase difference or increasing ellipticity monotonically lowers ɡ, reaching a local maximum of −0.93 at 798 nm for $\left|\phi_{s}-\phi_{p}\right|=15{}^{\circ}$. At the wavelength of 842 nm, one sees a similar trend in ɡ ranging from −2 for CPL to −0.52 for elliptically polarized light with $\left|\phi_{s}-\phi_{p}\right|=15{}^{\circ}$.

**FIGURE 3:**
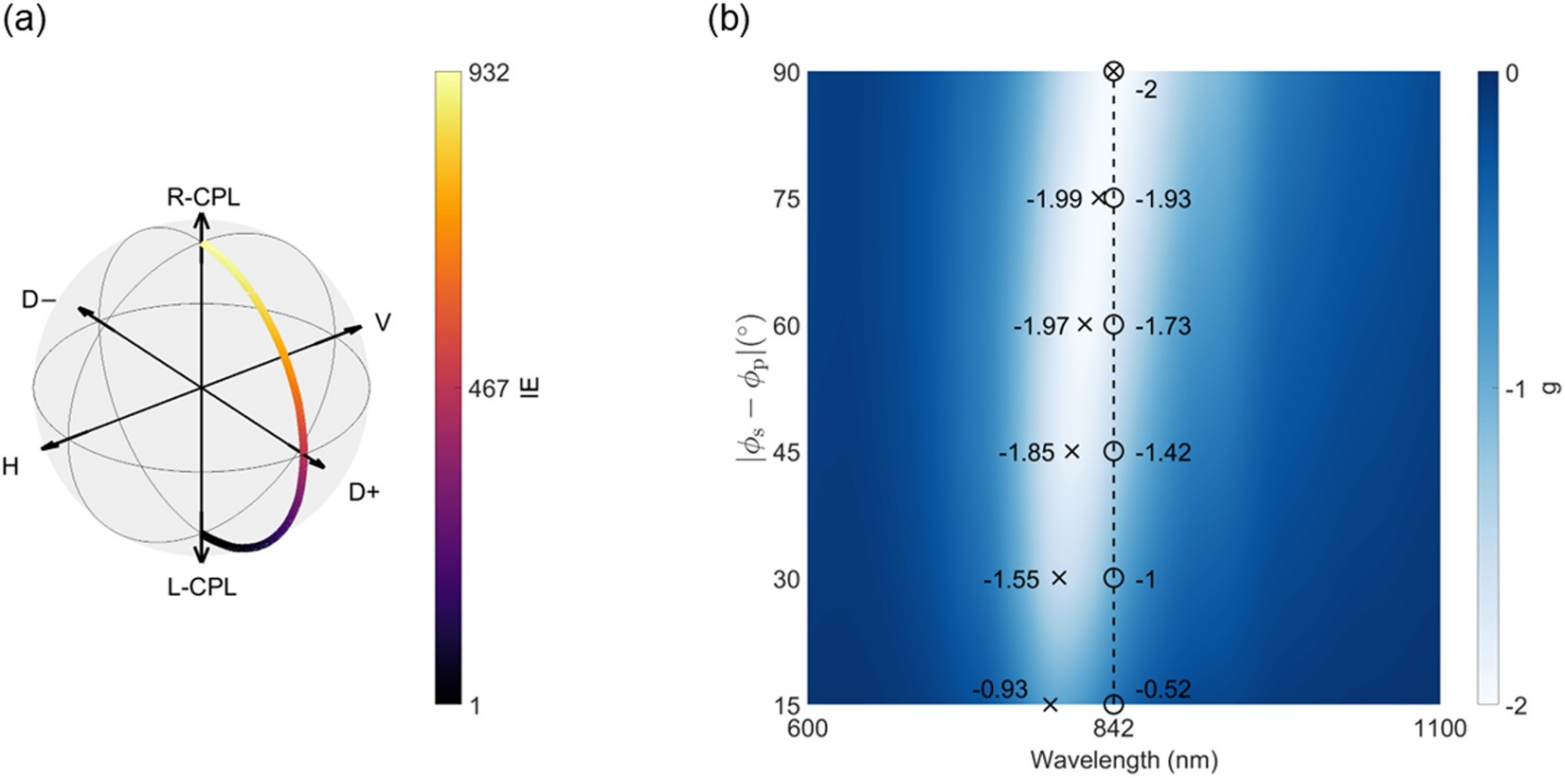
(a) Poincaré sphere representation of IE in the nanogap at the wavelength of 842 nm showing continuous modulation with varying excitation polarization state. A trajectory from R‐CPL to L‐CPL via D+ polarization yields continuous and near‐complete tunability, with IE peaking at 931.68 (approximated to 932) under R‐CPL and decreasing monotonically to 0.98 (approximated to 1) under L‐CPL. This corresponds to a modulation depth of approximately 100%. (b) ɡ as a function of excitation ellipticity, parameterized by the phase difference $\left|\phi_{s}-\phi_{p}\right|$. Maximal overall ɡ of approximately −2 is seen at 842 nm under CPL $\left(\left|\phi_{s}-\phi_{p}\right|=90{}^{\circ}\right)$, whereas reduced phase difference lowers ɡ (denoted by ×) monotonically to −0.93 at 798 nm for $\left|\phi_{s}-\phi_{p}\right|=15{}^{\circ}$. At 842 nm (vertically dashed line), ɡ (denoted by ○) decreases from −2 (CPL) to −0.52 $\left(\left|\phi_{s}-\phi_{p}\right|=15{}^{\circ}\right)$.

## 5 Far‐Field Response of Single‐Emitter‐Driven Planar Chiral Nanoantenna

Building on the demonstrated excitation‐chirality‐controlled hot spot switching, reciprocity dictates that the fully asymmetric nanoantenna should also impart spin selectivity to radiation from an achiral emitter coupled to the hot spot. Figure 4a exhibits far‐field 〈DoCP〉 from a single x‐polarized dipole source located at the center of the nanogap. One can consider this dipole to be mimicking a single emitter in the nanogap. DoCP is the ratio of the Stokes parameters S_3_ and S_0_ [38, 39, 40] and it can be mathematically described as follows,

(2) $$\begin{array}{c} \text{DoCP}\left(\theta ,\phi \right)=\frac{S_{3}\left(\theta ,\phi \right)}{S_{0}\left(\theta ,\phi \right)}=\frac{I\left\{E_{\theta }\left(\theta ,\phi \right)E_{\phi }^{*}\left(\theta ,\phi \right)-E_{\phi }\left(\theta ,\phi \right)E_{\theta }^{*}\left(\theta ,\phi \right)\right\}}{E_{\theta }\left(\theta ,\phi \right)E_{\theta }^{*}\left(\theta ,\phi \right)+E_{\phi }\left(\theta ,\phi \right)E_{\phi }^{*}\left(\theta ,\phi \right)} \end{array},$$

$E_{\theta }$ and $E_{\phi }$ represent the complex electric fields in the polar and azimuthal directions in the far field, respectively. 〈DoCP〉 is calculated by dividing the integrated DoCP recorded in the far field across the numerical aperture (NA) of 0.9 by the surface area projected by the same NA's light cone. In Figure 4a, 〈DoCP〉 is positive (blue) across the spectrum on the substrate side and negative (red) on the air side, highlighting broadband spin‐selective emission enabled by the nanoantenna's planar chiral configuration. At 842 nm (vertical dashed line), the nanoantenna exhibits high 〈DoCP〉 of 0.86 on the substrate side and −0.99 on the air side over an NA of 0.9. Figure 4b,c present the normalized radiated intensity pattern at 842 nm on the substrate and air sides, respectively. The polarization ellipses overlaid on the angular radiation patterns confirm that the polarization state on each side preserves highly spin‐selective behavior across the entire NA of 0.9. As shown in Supporting Information S1: Figure S8, the radiated intensity is stronger in the substrate than in air. The contrast in intensity arises because the higher index of substrate compared to that of air facilitates enhanced coupling to the propagating modes on the substrate side [41]. Since the dipole considered in Figure 4 is ideally configured with a position at the geometric center of the nanogap and an orientation along the width of the nanogap (x‐axis), the effect of variations in emitter position and dipole orientation is analyzed in the Supporting Information (Supporting Information S1: Figure S9) to further assess the robustness of the emitter‐driven far‐field response.

**FIGURE 4:**
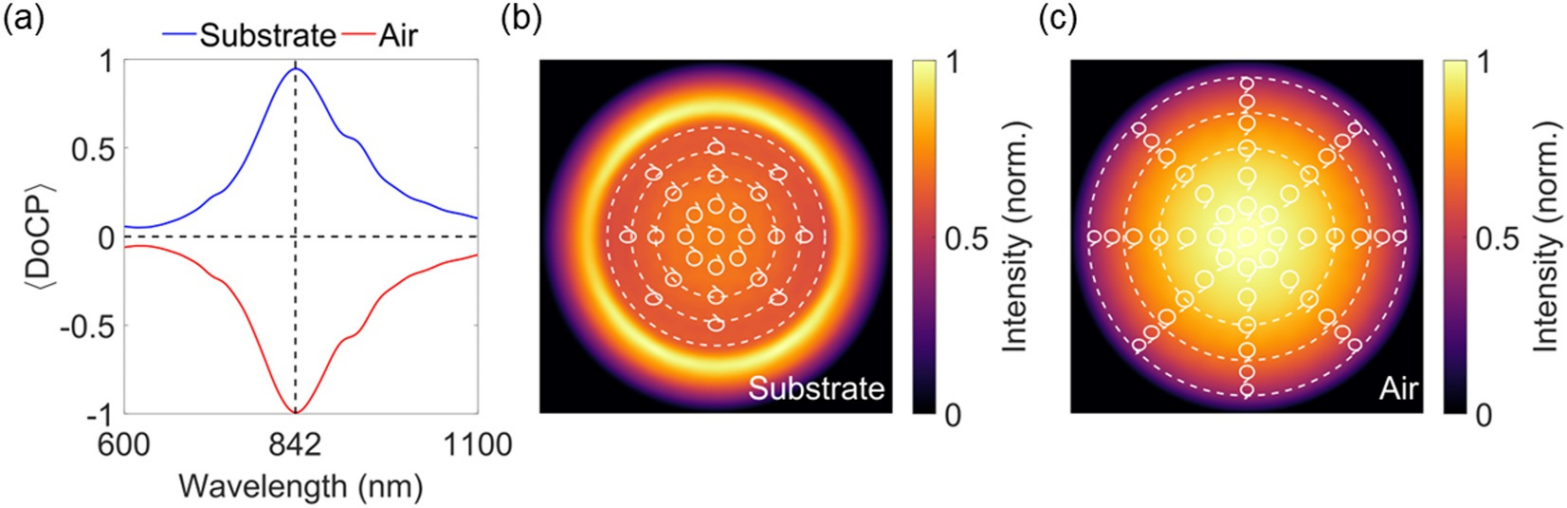
(a) Average degree of circular polarization (〈DoCP〉) in the far field calculated across a numerical aperture (NA) of 0.9 from an x‐polarized dipole emitter placed at the geometrical center of the planar chiral nanoantenna's nanogap. 〈DoCP〉 remains positive (blue) on the substrate side and negative (red) on the air side across the spectrum with its maximum values reaching 0.86 and −0.99 at 842 nm (vertical dashed line) on the substrate and air sides, respectively. Normalized radiation patterns at 842 nm on the (b) substrate and (c) air sides with overlaid polarization ellipses. The three dashed circular rings in both plots exhibit the NA, where the innermost to outermost rings correspond to NA = 0.5, 0.7, and 0.9, respectively.

## 6 Conclusion

In summary, we theoretically demonstrate a rationally designed planar chiral plasmonic nanoantenna that supports excitation‐chirality‐dependent modulation of hot spot and emitter‐coupled far‐field polarization engineering. The nanoantenna's hot spot intensity exhibits maximal ɡ (−2), complete modulation depth (100%), and very high ΔIE (930.7). This presents opportunities for diverse switchable or tunable applications in nanoscale optoelectronics, polarimetry, nonlinear optics, etc. We also show that coupling a linearly polarized achiral emitter to the nanogap yields radiation with near‐maximal 〈DoCP〉 (–0.99) over a large angular range, enabling deterministic generation of CPL. The high polarization purity and 〈DoCP〉 over an NA of 0.9, together with circularly polarized emission into both half‐spaces, establish the planar chiral nanoantenna as a robust platform for quantum‐emitter‐driven, spin‐resolved far‐field applications, such as chiral single‐photon sources. A quantitative comparison of the proposed planar chiral nanoantenna with representative state‐of‐the‐art nano‐optical platforms in terms of appropriate figures of merit such as ɡ, ΔIE, and 〈DoCP〉 is provided in the Supporting Information (Section 6).

The interference of spectrally broad modes of the planar chiral nanoantenna is robust to variations in fabricated nanostructure dimensions and experimental conditions, thus making the proposed nanoantenna design particularly suitable for future experimental work. Importantly, the design principles discussed here are materially agnostic and rely primarily on modal interference with optimized relations in location, polarization, amplitude, and phase. Therefore, the design criteria are universal and can be employed to discover and optimize other nanoantenna geometries with tailored chiral responses.

## 7 Methods

### 7.1 Numerical Electrodynamic Simulations

Numerical simulations are conducted using the FDTD algorithm within a commercial 3D electromagnetic solver (Lumerical 2024 R1.1, Ansys). Total‐field scattered‐field plane‐wave source at normal incidence is used to create the p‐polarized, s‐polarized, L‐CPL, and R‐CPL excitations. The nanoantennas are excited from the substrate side. For the far‐field polarization analysis, an x‐polarized dipole source is placed at the center of the nanoantenna's nanogap. The fields radiated from the dipole are recorded on the surface of a sphere of radius 1 m with the nanoantenna located at the sphere's center. Each hemisphere corresponds to one half‐space, with the upper half considered as air and the lower half as glass substrate. Far‐field quantities radiated from the dipole are calculated considering NA 0.9 in both half‐spaces. A spatial mesh resolution of 1 nm is employed to encompass the nanoantenna, with a refined mesh resolution of 0.5 nm applied in the nanogap region. Mesh convergence is evaluated by tracing the change in IE_R‐CPL_ and IE_L‐CPL_ spectra at the center of the nanogap under progressively refined nanogap mesh resolution ranging from 1 nm to 0.2 nm with the surrounding mesh fixed at 1 nm. The nanogap mesh resolution of 0.5 nm is adopted as a practical compromise between numerical accuracy and computational feasibility. Perfectly matched layer boundary conditions are used in all directions to eliminate spurious reflections. The simulation time and the built‐in auto‐shutoff criterion are chosen to ensure complete decay of the fields. All calculations in this work consider the monocrystalline gold permittivity from Olmon et al. [42] for the nanoantenna and the glass permittivity from Palik [43] for the substrate.

## Author Contributions

**Abhik Chakraborty:** conceptualization, investigation, writing – original draft, methodology, validation, visualization, writing – review and editing, software, formal analysis, data curation. **Xiaofei Wu:** writing – review and editing. **Ankit Kumar Singh:** writing – review and editing. **Fabian Scheidler:** writing – review and editing. **Min Jiang:** visualization, writing – review and editing. **Jürgen Popp:** funding acquisition, writing – review and editing. **Bert Hecht:** writing – review and editing. **Jer‐Shing Huang:** funding acquisition, writing – review and editing, supervision, resources, project administration.

## Funding

The work is funded by the Deutsche Forschungsgemeinschaft (DFG, German Research Foundation) via CRC/SFB 1375 NOA (project no. 398816777; sub‐project C1) and IRTG 2675 “Meta‐Active” (project no. 437527638; sub‐project C1).

## Conflicts of Interest

The authors declare no conflicts of interest.

## Supporting information


Supporting Information S1

## Data Availability

The data that support the findings of this study are available from the corresponding author upon reasonable request.
