# Supplementary material for: Planar Chiral Nanoantenna for Excitation‐Chirality‐Controlled Hot Spot Modulation and Emitter‐Coupled Circularly Polarized Emission
Source: Nanophotonics. 2026 Jul 14;15(14):e70199. doi: 10.1002/nap2.70199 (PMC13642169; doi:10.1002/nap2.70199)
Supplement: Supplementary file 1 [file NAP2-15-e70199-s001.pdf]

Supporting Information

Planar Chiral Nanoantenna for Excitation-Chirality-Controlled Hot Spot Modulation and Emitter-Coupled Circularly Polarized Emission

Abhik Chakraborty,[1,2,3,*] Xiaofei Wu,[3] Ankit Kumar Singh,[3] Fabian Scheidler,[4] Min Jiang,[2,3,5] Jürgen Popp,[1,2,3] Bert Hecht,[4] and Jer-Shing Huang[1,2,3,6,7,*]

[1]Institute of Physical Chemistry, Friedrich Schiller University Jena, Jena, Germany
[2]Abbe Center of Photonics, Friedrich Schiller University Jena, Jena, Germany
[3]Leibniz Institute of Photonic Technology, Jena, Germany
[4]Experimental Physics 5, University of Würzburg, Würzburg, Germany
[5]ARC Centre of Excellence for Transformative Meta-Optical Systems, Department of Electronic Materials Engineering, Research School of Physics, The Australian National University, Canberra, Australia
[6]Research Center for Applied Sciences, Academia Sinica, Taipei, Taiwan
[7]Department of Electrophysics, National Yang Ming Chiao Tung University, Hsinchu, Taiwan

*Corresponding authors: abhik.chakraborty@uni-jena.de, jer-shing.huang@leibniz-ipht.de

I. Study of the Planar Chiral Nanoantenna's Near-Field Response with Variation of Structural Parameters

II. Study of the Planar Chiral Nanoantenna's Near-Field Response with Variation of Illumination Parameters

III. Modal Analysis of Fully Symmetric, Partially Symmetric, and Planar Chiral Nanoantennas

IV. Radiated Intensity Spectra of Single-Emitter-Driven Planar Chiral Nanoantenna

V. Effect of Emitter Position and Orientation on the Far-Field Response of the Planar Chiral Nanoantenna

VI. Comparative Evaluation of the Proposed Planar Chiral Nanoantenna with Representative Nano-Optical Platforms

I. Study of the Planar Chiral Nanoantenna's Near-Field Response with Variation of Structural Parameters

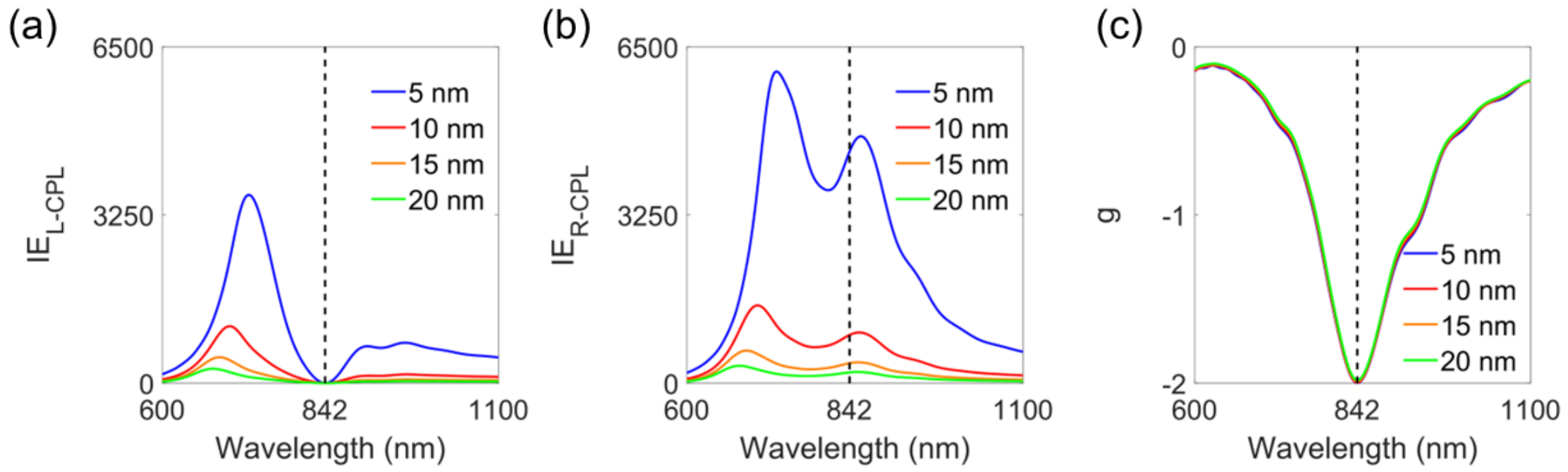

Figure S1. Intensity enhancement (IE) spectra at the geometric center of the plasmonic nanogap under left-handed circularly polarized light (L-CPL) and right-handed circularly polarized light (R-CPL) excitation defined as (a) $IE_{L\text{-}CPL}$ and (b) $IE_{R\text{-}CPL}$, respectively, for varying nanogap widths (W) of 5 nm, 10 nm, 15 nm, and 20 nm. (c) Corresponding dissymmetry factor (g) spectra exhibits maximal value at 842 nm as approximately −2.00, −2.00, −1.99, and −1.98 for W = 5 nm, 10 nm, 15 nm, and 20 nm, respectively.

To evaluate the influence of the nanogap width (defined as W in **Figure 1a** in the main manuscript) on the chiroptical near-field response, the planar chiral nanoantenna is simulated for W = 5 nm, 10 nm, 15 nm, and 20 nm. The near-field response is monitored at the geometric center of the nanogap under left-handed circularly polarized light (L-CPL) and right-handed circularly polarized light (R-CPL) excitation at normal incidence from the substrate side. **Figure S1a** and Figure S1b show that under L-CPL and R-CPL excitation, increasing W progressively reduces the near-field intensity enhancement $IE_{L\text{-}CPL}$ and $IE_{R\text{-}CPL}$, respectively, due to the weaker electromagnetic confinement within the nanogap. However, the overall spectral characteristics remain largely unchanged for both. Figure S1c exhibits that the near-field dissymmetry factor g $\left(\frac{IE_{L-CPL}-IE_{R-CPL}}{IE_{L-CPL}+IE_{R-CPL}} \times 2\right)$ spectra at the geometric center of the nanogap remains unaffected by the change in W. Notably, all investigated cases exhibit their maximal g at the same wavelength of 842 nm, with its values being approximately −2.00, −2.00, −1.99, and −1.98 for W = 5 nm, 10 nm, 15 nm, and 20 nm, respectively. This demonstrates that the underlying excitation-chirality-dependent modal interference responsible for the chiral switching behavior is robust against variations in the nanogap size.

Furthermore, a quantitative spectral analysis of IE at the geometric center of the nanogap has been performed as a function of the constituent nanorod lengths L_1 and L_2 (as defined in Figure 1a in the main manuscript) of each V-shaped element. This is achieved by varying one nanorod length L_1 from 72 nm to 252 nm in steps of 20 nm while keeping the other nanorod length L_2 constant. The resulting variation in the planar chiral nanoantenna's near-field response is systematically investigated for two representative values of L_2 (112 nm and 152 nm). **Figure S2a** and Figure S2b show $IE_{R\text{-}CPL}$ and $IE_{L\text{-}CPL}$, respectively, for L_2 = 112 nm, while Figure S2c and Figure S2d show $IE_{R\text{-}CPL}$ and $IE_{L\text{-}CPL}$, respectively, for L_2 = 152 nm. In all cases, distinct modal branches emerge, with their spectral positions represented in wavelength varying with the overall cavity length ($L_1 + L_2$) and their IE governed by the phase relationship associated with L-CPL and R-CPL illumination. In Figure S2a and Figure S2b, the red × marks the proposed planar chiral nanoantenna geometry (L_1 = 92 nm, L_2 = 112 nm) at and around the operating wavelength of 842 nm. As expected, a pronounced contrast in IE is observed in this

region, with $IE_{R\text{-}CPL}$ exhibiting a bright hotspot and $IE_{L\text{-}CPL}$ exhibiting a strongly suppressed response.

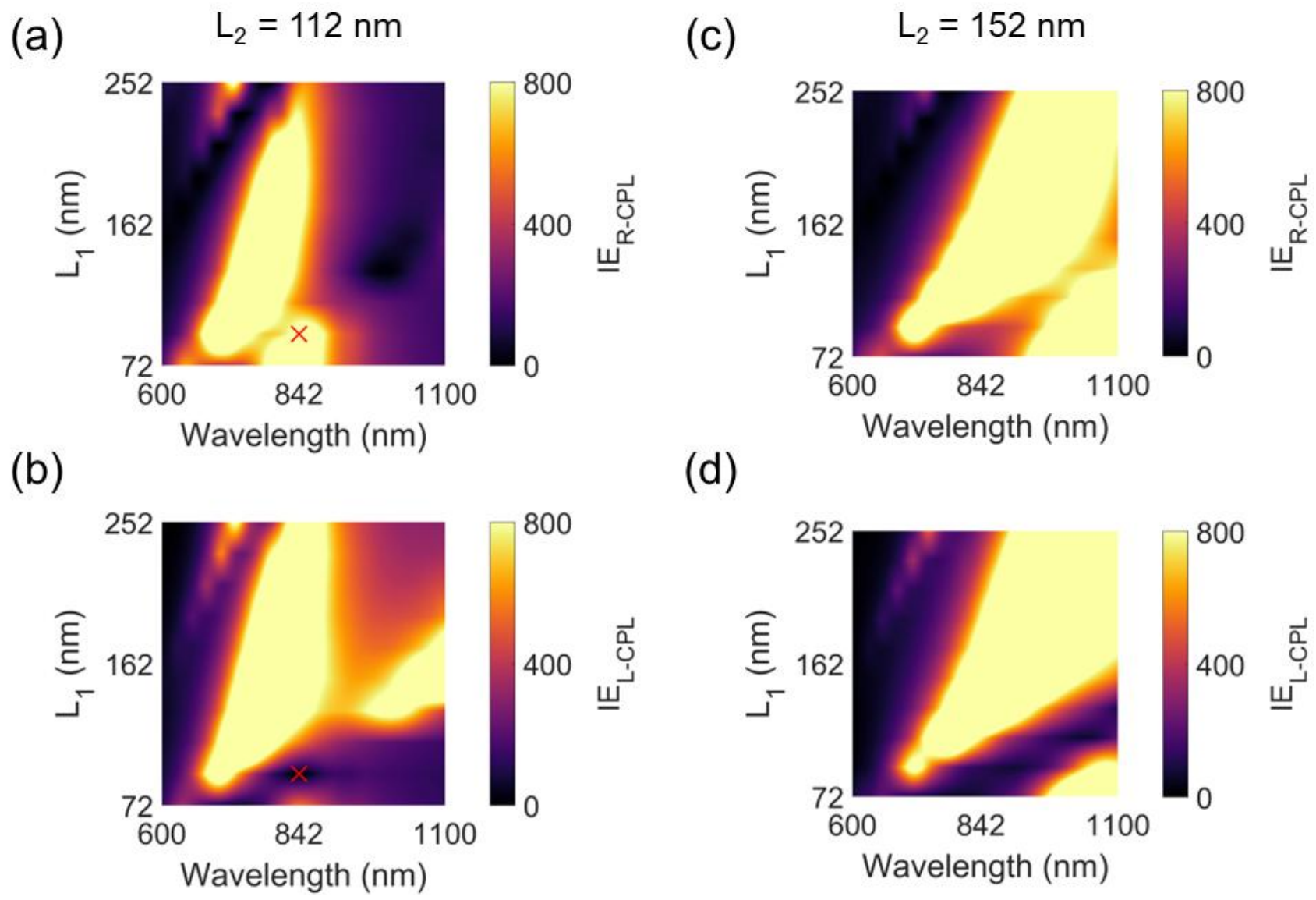

Figure S2. Spectral dependence of $IE_{R\text{-}CPL}$ and $IE_{L\text{-}CPL}$ at the geometric center of the nanogap as a function of constituent nanorod length L_1 ranging from 72 nm to 252 nm and two representative values of L_2 = 112 nm and 152 nm. (a) $IE_{R\text{-}CPL}$ and (b) $IE_{L\text{-}CPL}$ for L_2 = 112 nm. (c) $IE_{R\text{-}CPL}$ and (d) $IE_{L\text{-}CPL}$ for L_2 = 152 nm. In all cases, multiple modal branches are observed, with their spectral positions varying as a function of the overall cavity length $L_1 + L_2$ and their IE determined by phase relationships imposed by the handedness of excitation. The red × in (a) and (b) marks the planar chiral nanoantenna geometry investigated in the main manuscript (L_1 = 92 nm, L_2 = 112 nm) at the operating wavelength of 842 nm, where a pronounced contrast between $IE_{R\text{-}CPL}$ and $IE_{L\text{-}CPL}$ is observed.

To further identify regions exhibiting strong excitation-chirality-controlled switching, the corresponding g and absolute near-field intensity contrast ($\Delta IE = IE_{R\text{-}CPL} - IE_{L\text{-}CPL}$) were evaluated at the geometric center of the nanogap for the same parameter space. **Figure S3a** and Figure S3b show the resulting g and ΔIE maps, respectively, for L_2 = 112 nm, while Figure S3c and Figure S3d depict the corresponding maps for L_2 = 152 nm. As expected from the IE maps presented in Figure S2, several regions exhibiting simultaneously large values of |g| and |ΔIE| are observed.

As expected, L_1 = 92 nm and L_2 = 112 nm demonstrates maximal g of −2 and very high ΔIE of 930.7 at the wavelength of 842 nm (marked by ×) in Figure S3a and Figure S3b, respectively. For L_1 = 132 nm and L_2 = 112 nm, a particularly strong response is obtained at 993 nm (marked by ○), where g = 1.98 in Figure S3a and ΔIE = −913.76 in Figure S3b approximately. Likewise, for L_1 = 112 nm and L_2 = 152 nm, a strong response is obtained at 1044 nm (marked by △), where g = −1.79 in Figure S3c and ΔIE = 1053.17 in Figure S3d approximately. These results confirm that the excitation-chirality-dependent switching mechanism is not unique to the geometry investigated in the main manuscript but can also be realized for alternative cavity dimensions and operating wavelengths. To quantify the extent of the parameter space supporting strong switching behavior, threshold values of $|g| \geq 1.75$ and $|\Delta IE| \geq 800$ are imposed. Contiguous regions satisfying both criteria are identified for L_2 = 112 nm, spanning L_1 from 77 nm to 95 nm at wavelengths around 842 nm in one case and L_1 from 129 nm to 155 nm at wavelengths surrounding 993 nm in another case. Similarly, a contiguous region satisfying the same pair of criteria is identified for L_2 = 152 nm, spanning L_1 from 108 nm to 115 nm at wavelengths around 1044 nm. The apparent continuity of these regions reflects

interpolation between discretely sampled data points (ΔL_1 = 20 nm and ΔL_2 = 40 nm), suggesting that finer sampling would likely smoothen these domains. The existence of these extended regions indicates that the excitation-chirality-controlled switching mechanism does not rely on a singular geometrical configuration but rather persists over a finitely extended design parameter space, thereby allowing sizable tolerance regions for fabrication errors that might deviate realized structural parameters from intended structural parameters of the planar chiral nanoantenna. Given the limited number of data points (L_1 = 92 nm − 152 nm, L_2 = 112 nm and 152 nm, and wavelength = 600 nm − 1100 nm) employed in the present study keeping computational economics in mind, the identified data points and regions of optimal or near-optimal performance likely imply a larger possible parameter space supporting strong switching behavior.

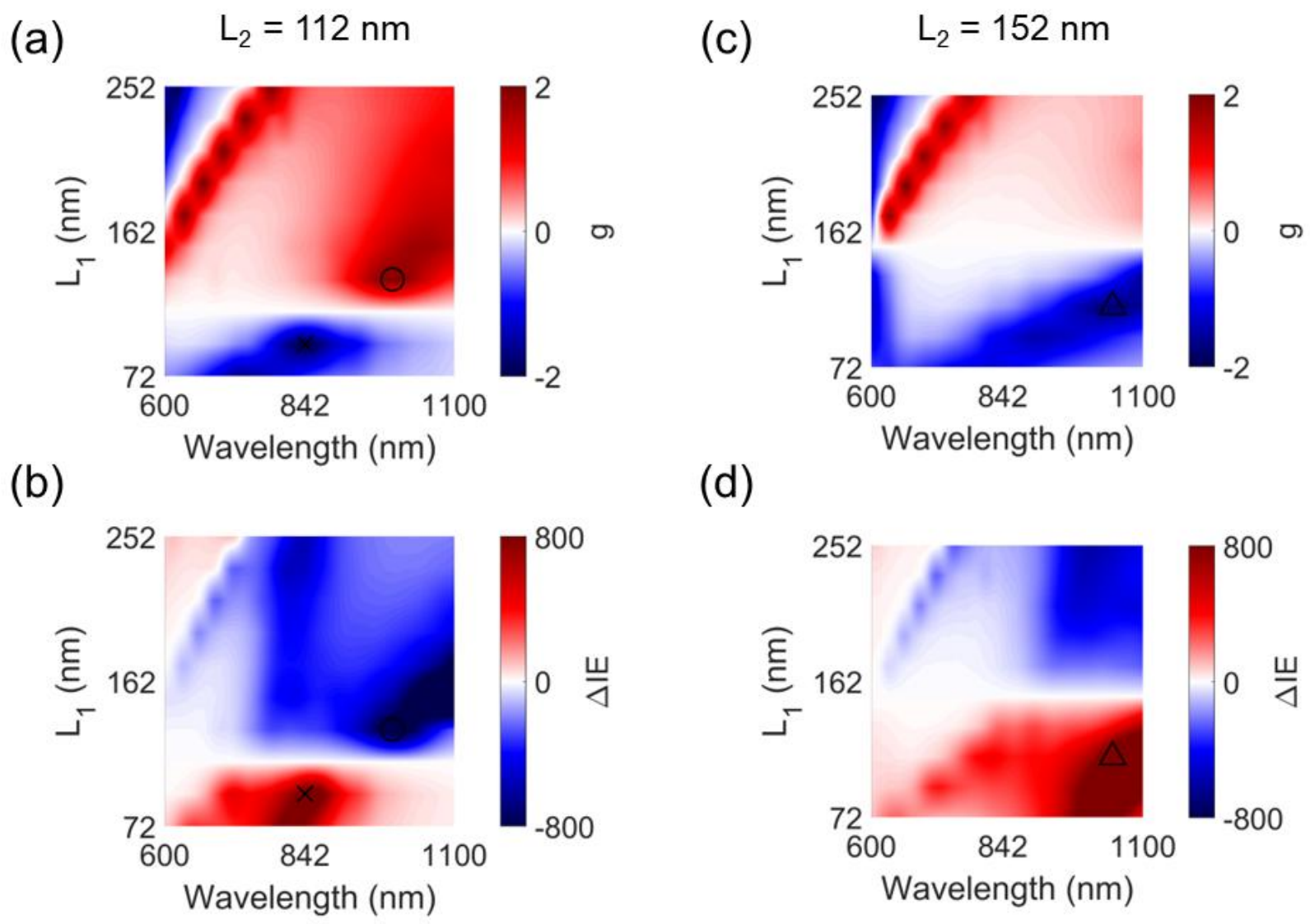

Figure S3. Spectral dependence of g and intensity contrast (ΔIE) at the geometric center of the nanogap as a function of constituent nanorod length L_1 ranging from 72 nm to 252 nm and two representative values of L_2 = 112 nm and 152 nm. (a) g and (b) ΔIE for L_2 = 112 nm. (c) g and (d) ΔIE for L_2 = 152 nm. The black × symbol in (a) and (b) marks the nanoantenna geometry investigated in the main manuscript (L_1 = 92 nm, L_2 = 112 nm) at the wavelength of 842 nm. The black ○ symbol in (a) and (b) and the black △ symbol in (c) and (d) indicate data points representing alternative geometries and operating wavelengths exhibiting strong excitation-chirality-dependent switching behavior.

II. Study of the Planar Chiral Nanoantenna's Near-Field Response with Variation of Illumination Parameters

To account for the planar chiral nanoantenna's tolerance to alignment errors and accidental tilts in the illumination, $IE_{L\text{-}CPL}$, $IE_{R\text{-}CPL}$, and g are simulated at the geometric center of the nanogap under tilted incidence from the substrate side with angle of incidence to the surface normal defined as η = 10° and 20° (**Figure S4a**). We believe that the chosen values of η comfortably outstrip any possible alignment error and accidental tilt in incidence. Moreover, since tilted CPL illumination appears elliptically polarized to the sample plane and the nanoantenna itself is asymmetric in its in-plane geometry, the nanoantenna is rotated by angle ζ = 0°, 45°, and 90° to the x-axis (Figure S4b) to make sure that it interacts with each case of tilted incidence (η = 10° and 20°) at different in-plane orientations. Consequently, the near-field response for each combination of η and ζ is quantified. Keeping computational economics in mind, the simulations are executed within a limited wavelength range of 802 nm to 882 nm with a regular interval of 10 nm.

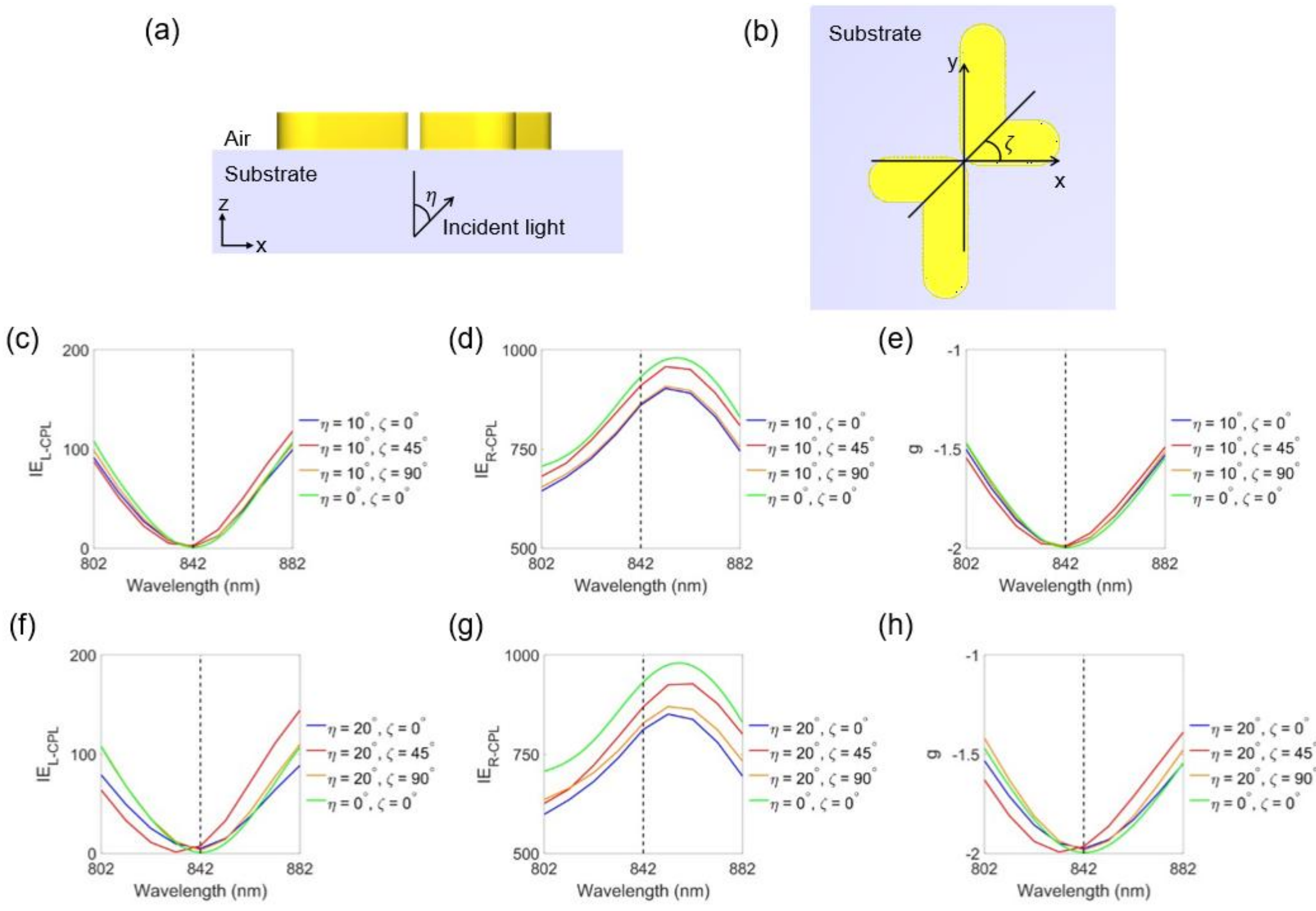

Figure S4. (a) Schematic of tilted incidence from the substrate side, where η denotes the angle of incidence with respect to the surface normal. (b) Schematic of the in-plane rotated planar chiral nanoantenna with the rotation angle ζ defined relative to the x-axis. (c,d) $IE_{L\text{-}CPL}$ and $IE_{R\text{-}CPL}$ spectra, respectively, at the geometric center of the nanogap for η = 10° and ζ = 0°, 45°, and 90°. (e) Corresponding g spectra for η = 10° and ζ = 0°, 45°, and 90°. (f,g) $IE_{L\text{-}CPL}$ and $IE_{R\text{-}CPL}$ spectra, respectively, for η = 20° and ζ = 0°, 45°, and 90°. (h) Corresponding g spectra for η = 20° and ζ = 0°, 45°, and 90°. Spectra for the original operating condition (η = 0°, ζ = 0°) are included in (c−h) for comparison.

Figure S4c and Figure S4d exhibit $IE_{L\text{-}CPL}$ and $IE_{R\text{-}CPL}$ spectra, respectively, for η = 10° and ζ = 0°, 45°, and 90°. Corresponding spectra of g for η = 10° and ζ = 0°, 45°, and 90° are provided in Figure S4e. Similarly, Figure S4f and Figure S4g exhibit $IE_{L\text{-}CPL}$ and $IE_{R\text{-}CPL}$ spectra,

respectively, for $\eta = 20°$ and $\zeta = 0°$, 45°, and 90°. Corresponding spectra of g for $\eta = 20°$ and $\zeta = 0°$, 45°, and 90° are provided in Figure S4h. Additionally, $IE_{L\text{-}CPL}$ (Figure S4c, Figure S4f), $IE_{R\text{-}CPL}$ (Figure S4d, Figure S4g), and g (Figure S4e, Figure S4h) from the originally simulated condition of $\eta = 0°$ (normal incidence) and $\zeta = 0°$ (nanogap oriented along the x-axis) are provided for comparison. For $\eta = 10°$, g records maximal dissymmetry at values like −1.99 ($\zeta = 0°$, wavelength 842 nm), −1.99 ($\zeta = 45°$, wavelength 842 nm), and −2 ($\zeta = 90°$, wavelength 842 nm). For $\eta = 20°$, g records maximal dissymmetry at values like −1.98 ($\zeta = 0°$, wavelength 842 nm), −1.99 ($\zeta = 0°$, wavelength 832 nm), and −1.98 ($\zeta = 0°$, wavelength 842 nm). All the results agree strongly with each other and with the optimal condition of g = −2 at 842 nm for $\eta = 0°$, $\zeta = 0°$.

Due to the relatively coarse wavelength resolution of 10 nm employed in the simulations, the wavelengths corresponding to the maximal dissymmetry will not necessarily coincide with the true optimal values. Nevertheless, the observed data points calculated under the different illumination conditions remain in close agreement with each other and the ideal operating condition ($\eta = 0°$, $\zeta = 0°$) of the planar chiral nanoantenna. This confirms a high degree of robustness against illumination tilt.

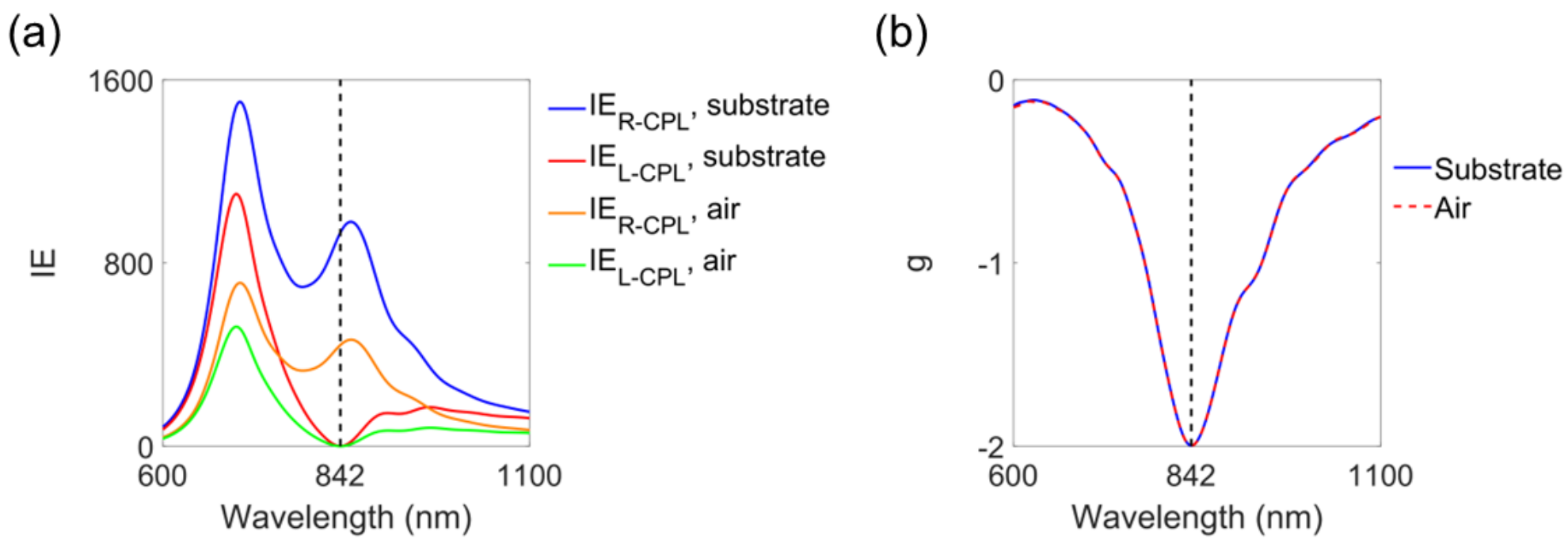

Figure S5. (a) $IE_{R\text{-}CPL}$ and $IE_{L\text{-}CPL}$, at the geometric center of the nanogap for normal incidence from the substrate side and from the air side. While the absolute values of the intensity enhancement are higher for substrate-side illumination, the spectral characteristics remain unchanged. (b) Corresponding g spectra for substrate-side and air-side illumination. Both illumination directions exhibit highly coinciding g spectra with maximal dissymmetry characterized by g = −2 at 842 nm.

Beside angular tilts, the stability of the planar chiral nanoantenna's near-field response spectra ($IE_{L\text{-}CPL}$, $IE_{R\text{-}CPL}$, and g) is tested at the geometric center of the nanogap under normally incident excitation with two different directions of illumination, i.e., illumination from the substrate side and that from the air side. **Figure S5a** shows that both $IE_{R\text{-}CPL}$ and $IE_{L\text{-}CPL}$ spectra register higher values for incidence from the substrate side in comparison to that for incidence from the air side. However, the spectral shape of IE for each handedness of CPL remains the same for the two illumination directions. Moreover, the robustness of the planar chiral nanoantenna's switchability under the two illumination directions is tested by comparing the g spectra corresponding to substrate-side illumination with the g spectra corresponding to air-side illumination in Figure S5b. The two cases provide highly coinciding spectra and register maximal dissymmetry with g = −2 at 842 nm wavelength. The agreement in the relative contrast of IE for the two cases confirms the stability of the planar chiral nanoantenna's near-field response against changes in illumination direction.

III. Modal Analysis of Fully Symmetric, Partially Symmetric, and Planar Chiral Nanoantennas

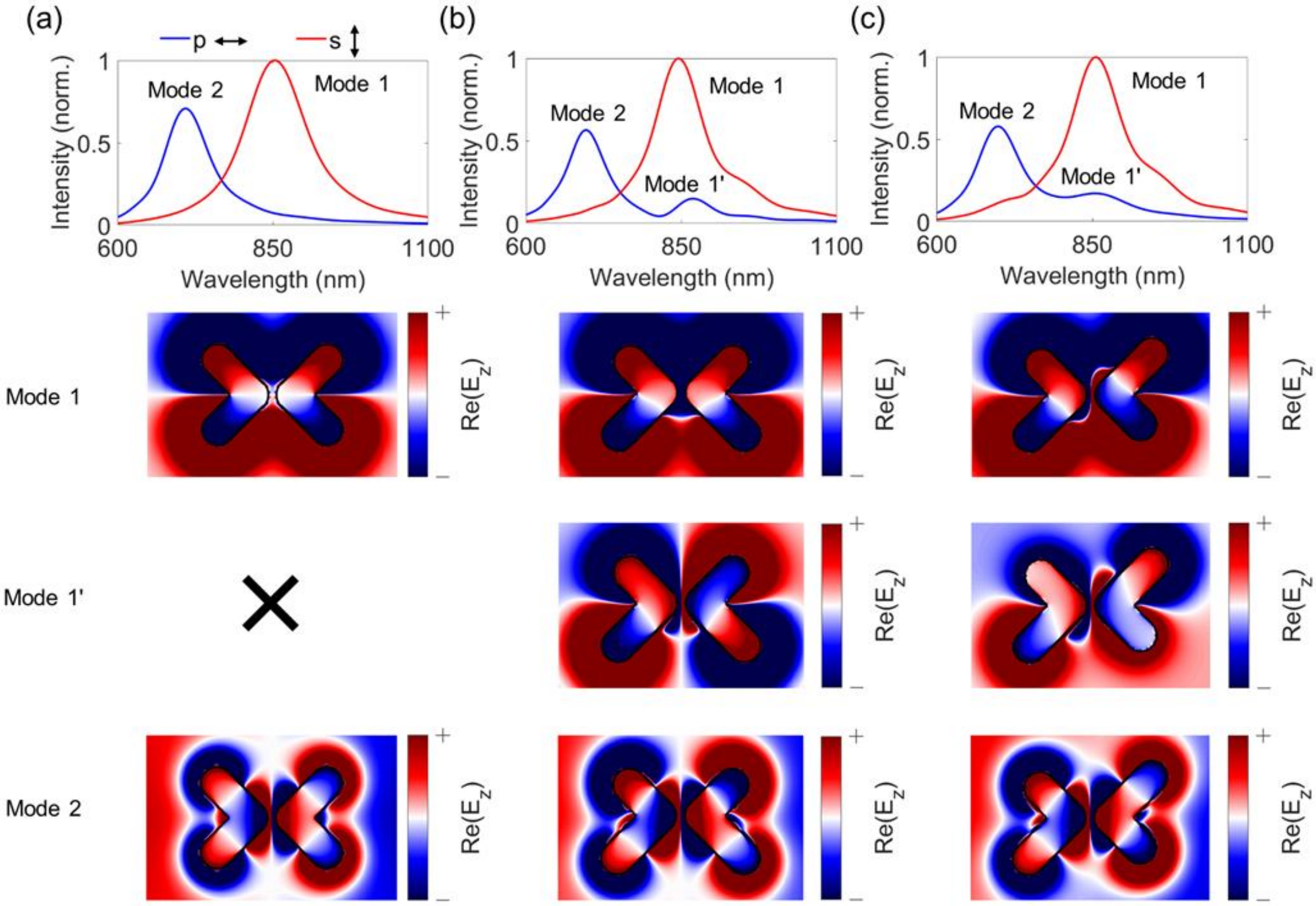

Figure S6. Scattering spectra and modal field distributions ($\mathrm{Re}(E_z)$) of the (a) fully symmetric (left column), (b) partially symmetric (middle column), and (c) planar chiral (right column) nanoantennas. The first row (top to bottom perspective) shows the normalized scattering spectra of (a) fully symmetric, (b) partially symmetric, and (c) planar chiral nanoantennas under p- (blue) and s-polarized (red) excitation. Mode 1 is the s-excited fundamental mode, Mode 1' is the p-excited fundamental mode, and Mode 2 is the p-excited higher-order mode. The second row exhibits the field distributions associated with Mode 1 in (a) fully symmetric, (b) partially symmetric, and (c) planar chiral nanoantennas. The third row exhibits the modal field distributions associated with Mode 1' in (b) partially symmetric and (c) planar chiral nanoantennas. The fourth row depicts the field distributions associated with Mode 2 in (a) fully symmetric, (b) partially symmetric, and (c) planar chiral nanoantennas.

To elucidate the origin of chirality, three nanoantenna geometries, i.e., fully symmetric (**Figure S6a**; left column of Figure S6), partially symmetric (Figure S6b; middle column), and planar chiral (Figure S6c; right column), are analyzed. The first row (top to bottom perspective) of Figure S6 depicts the normalized scattering spectra and dominant modes of the fully symmetric (Figure S6a), partially symmetric (Figure S6b), and planar chiral (Figure S6c) nanoantennas under p- (blue) and s-polarized (red) plane-wave excitation at normal incidence. p denotes the linear polarization of the excitation that is oriented along the axis of the nanoantenna's gap, whereas s denotes the excitation's linear polarization oriented orthogonally (in-plane) to the same axis. Here, the s-excited fundamental mode is marked as Mode 1, the p-excited fundamental mode is marked as Mode 1', and the p-excited higher-order mode is marked as Mode 2. The second row shows the field distributions ($\mathrm{Re}(E_z)$) associated with Mode 1 in fully symmetric (Figure S6a), partially symmetric (Figure S6b), and planar chiral (Figure S6c) nanoantennas. Similarly, the third row exhibits the field distributions ($\mathrm{Re}(E_z)$) associated with Mode 1' in partially symmetric (Figure S6b) and planar chiral (Figure S6c) nanoantennas.

Finally, the fourth row exhibits the field distributions ($\mathrm{Re}(E_z)$) associated with Mode 2 in fully symmetric (Figure S6a), partially symmetric (Figure S6b), and planar chiral (Figure S6c) nanoantennas.

Even though E_x is the key to understanding excitation-chirality-controlled dissymetry in the x-axis-oriented nanogap due to the nanogap's geometry-induced confinement (**Figure 2** in the main manuscript), the real part of the out-of-plane field component ($\mathrm{Re}(E_z)$) is used to study the modal field distribution at a plane intersecting each planar nanoantenna 1 nm below its surface (top air−metal interface) in Figure S6. This is done because the out-of-plane component provides an unambiguous representation of the modal field distributions whereas in-plane components ($\mathrm{Re}(E_x)$ and $\mathrm{Re}(E_y)$) exhibit mixed polarization contributions in these complex planar geometries.

By comparing the analysis of the chiroptical response depicted in Figure 2 of the main manuscript with the spectral position of the modes shown in Figure S6, one can deduce that the simultaneously p- and s-excited fundamental modes, i.e., Mode 1' and Mode 1 respectively, with equal amplitude and appropriate phase difference co-polarized and co-localized in the nanogap determines the pronounced chiroptical response in our work. Figure S6 shows that symmetry-related constraints prohibit the emergence of Mode 1' in the fully symmetric nanoantenna (Figure S6a; third row). Furthermore, the fully symmetric nanoantenna geometry supports a symmetric field distribution corresponding to Mode 1 with nodes at the apexes of the two symmetric V-shaped elements, thereby producing minimal to no amplitude enhancement (AE) in the nanogap. Therefore, we can exclude the fully symmetric nanoantenna (Figure S6a) and Mode 2 (Figure S6; fourth row) from the discussion and concentrate the analysis exclusively on Mode 1 and Mode 1' in the partially symmetric nanoantenna and the planar chiral nanoantenna.

Anti-symmetric field distributions corresponding to Mode 1' are present in both partially symmetric (Figure S6b; third row) and planar chiral (Figure S6c; third row) nanoantennas. The anti-symmetric field distributions allow the emergence of opposite polarity at the apexes of the partially symmetric and planar chiral nanoantennas under p-polarized excitation, thereby facilitating x-polarized AE in both nanogaps (Figure 2b and Figure 2c in the main manuscript). Therefore, the missing piece in the analysis is understanding the role of structural asymmetry in endowing the s-excited Mode-1-induced AE in the nanogap.

The partially symmetric nanoantenna geometry (Figure S6b; second row) supports a symmetric field distribution corresponding to Mode 1 in the two asymmetric V-shaped elements. Due to the asymmetric geometry of each V-shaped element in the partially symmetric nanoantenna, the node is slightly displaced from the apex. However, the symmetric field distribution results in a weakly coupled anti-bonding mode (same field polarity at the apexes) and minimal x-polarized AE in the nanogap (Figure 2b in the main manuscript). The planar chiral nanoantenna geometry (Figure S6c; second row), due to its asymmetric V-shaped elements having a mirror-inverted geometrical configuration with respect to each other, supports an anti-symmetric field distribution corresponding to Mode 1 in the two V-shaped elements, thereby yielding opposite polarity at the apexes of the V-shaped elements. This enforces a bonding mode and strong x-polarized AE within the nanogap (Figure 2c in the main manuscript). The anti-symmetric field distribution of Mode 1 is identical to that of Mode 1', which is unique to the planar chiral nanoantenna.

The emergence of Mode-1- and Mode-1'-induced x-polarized AE in the planar chiral nanoantenna underlines the role of complete planar asymmetry in inducing spatially overlapping and co-polarized electric near fields under linearly polarized orthogonal excitations. This, along with optimally engineered relations between the two modes' x-polarized AE (Figure 2c and Figure 2e in the main manuscript) and phase difference (Figure 2d and Figure 2f in the main manuscript), facilitate the emergence of chiroptical response in the planar chiral nanoantenna.

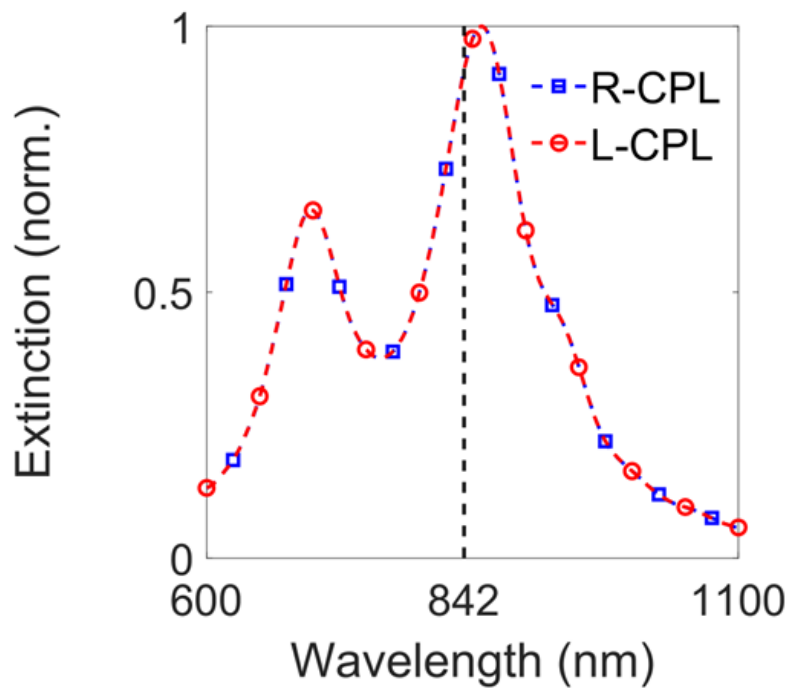

Figure S7. Normalized extinction spectra of the planar chiral nanoantenna under R-CPL and L-CPL illumination, showing no circular dichroism in the far field despite strong excitation-chirality-dependent near-field switching in the nanogap.

Interestingly, the maximal g observed in the planar chiral nanoantenna's near field is accompanied by no difference in the extinction spectra under R-CPL and L-CPL excitation (**Figure S7**). This behavior is consistent with the idea that the chiral response in this nanoantenna is governed primarily by excitation-chirality-dependent phase relationships between spatially overlapping plasmonic modes of equal AE rather than by differences in modal excitation efficiency or power dissipation. Under two opposite handedness of CPL, comparable plasmonic modes are excited with nearly identical AE in the planar chiral nanoantenna. However, the relative phase between these modal contributions changes under a switch in excitation chirality or handedness, thus modifying the interference condition within the nanoantenna, where one handedness concentrates the electromagnetic field in the nanogap and the other redistributes it over the surrounding nanoantenna. Since extinction measurements probe the total absorbed and scattered power rather than the local field distribution, this phase-driven near-field switching with near-maximal to maximal values of g in the near field remains largely hidden in the extinction response. In other words, the near-field chiroptical response in the planar chiral nanoantenna originates from excitation-chirality-dependent phase-controlled modal redistribution of electromagnetic fields rather than excitation-chirality-dependent differential interaction strengths.

IV. Radiated Intensity Spectra of Single-Emitter-Driven Planar Chiral Nanoantenna

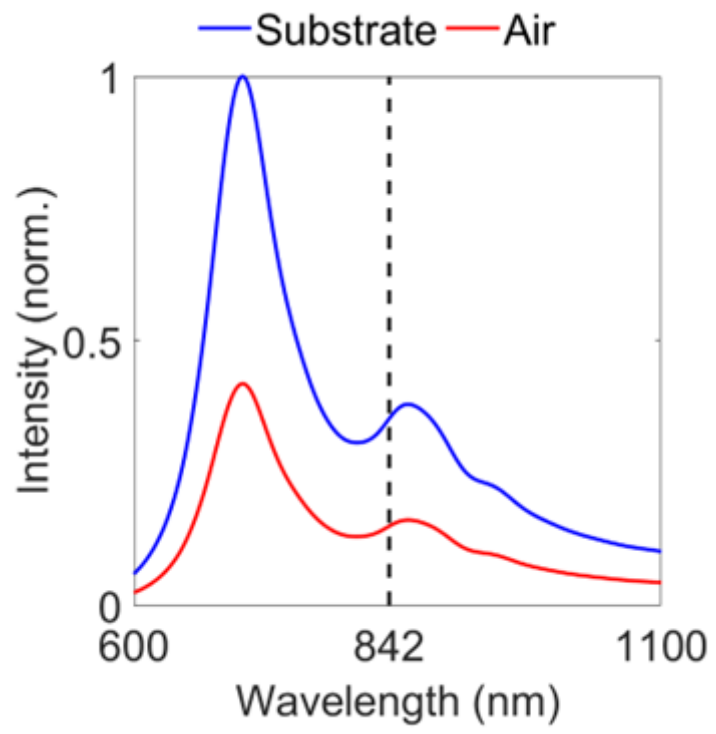

Figure S8. Normalized far-field intensity enhancement spectra radiated into substrate (blue) and air (red) directions from a dipole positioned at the geometric center of the planar chiral nanoantenna's nanogap and collected within a numerical aperture (NA) of 0.9.

Stokes parameters and, therefore, average degree of circular polarization (⟨DoCP⟩) are defined even in regions of space containing very low field. Therefore, while **Figure 4a** in the main manuscript exhibits very high ⟨DoCP⟩ values at and around 842 nm radiated into both the air side and the substrate side from an x-polarized dipole placed in the geometrical center of the planar chiral nanoantenna's nanogap, corresponding radiated intensity spectra are required to fully assess the efficiency of the dipole-in-nanoantenna system. **Figure S8** exhibits the normalized far-field intensity enhancement spectra of an x-polarized dipole placed in the nanoantenna's nanogap, with the nanoantenna situated on the substrate. The intensity radiated into a particular direction (substrate or air) is calculated within a numerical aperture (NA) of 0.9 and normalized by the intensity of the same dipole situated in free space radiated into the same direction and collected within the same NA. The spectra exhibited in Figure S8 are obtained by further normalizing the far-field intensity enhancement spectra by the highest value of radiated intensity recorded in the substrate. The wavelength of 842 nm (vertical dashed line) is marked by near-maximal ⟨DoCP⟩ in Figure 4a of the main manuscript, while also being very close to the fundamental modal peak of the nanoantenna's radiated intensity in Figure S8. This confirms the spectral region at and around 842 nm to be ideal for chiral emission from the optimally designed planar chiral nanoantenna. It is clear that the radiated intensity is higher into the substrate than that into air, with the intensity radiated into the substrate being 2.36 times that radiated into air at the wavelength of 842 nm.

V. Effect of Emitter Position and Orientation on the Far-Field Response of the Planar Chiral Nanoantenna

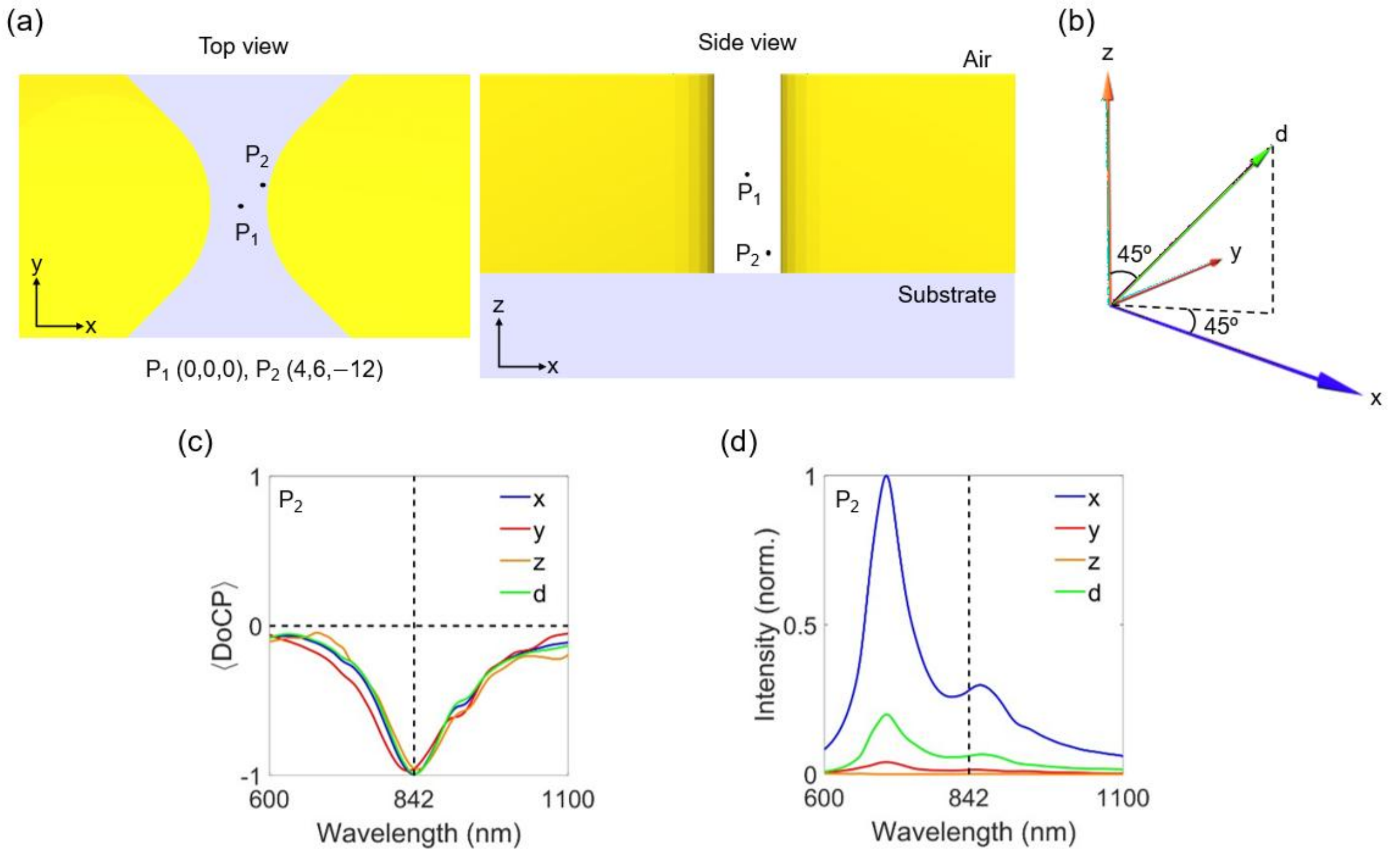

Figure S9. (a) Schematic illustration of the reference dipole position P_1 (0,0,0) at the geometric center of the nanogap and the displaced position P_2 (4,6,−12), shifted by +4 nm (x-axis), +6 nm (y-axis), and −12 nm (z-axis) relative to P_1. The left panel exhibits a top-view perspective while the right panel depicts a side-view perspective. The far-field response is evaluated for the displaced emitter position P_2. (b) Considered dipole orientations at P_2, including x-polarized (along the nanogap width W), y-polarized, z-polarized, and a diagonal orientation d at 45° with respect to the x-, y-, and z-axes. (c) Spectral variation of the average degree of circular polarization (⟨DoCP⟩) for each dipole orientation at P_2, evaluated on the air side within an NA of 0.9. (d) Normalized radiated intensity spectra on the air side within an NA of 0.9 for the same dipole configurations. The displaced emitter exhibits orientation-dependent radiated intensity due to variation in coupling efficiency to nanoantenna modes, while maintaining near-maximal ⟨DoCP⟩ across all orientations at wavelengths at and near the operating wavelength of 842 nm.

Figure 4 in the main manuscript considers an idealized dipolar emitter which is situated at the geometric center of the nanogap and is oriented along the x-axis or the width W of the nanogap. To assess the tolerance of the emitter-integrated planar chiral nanoantenna to uncertainties in emitter position and orientation, the far-field response is investigated for a dipolar emitter displaced from the geometric center of the nanogap. In **Figure S9a**, the reference emitter position P_1 (0, 0, 0) corresponds to the geometric center of the nanogap considered in the main manuscript, whereas the displaced emitter position P_2 is shifted by +4 nm along the x-axis, +6 nm along the y-axis, and −12 nm along the z-axis relative to P_1. The position P_2 (4, 6, −12) is arbitrarily chosen within the spatial constraints imposed by the nanogap dimensions. It is this displaced emitter whose far-field response is investigated. At P_2, four dipole orientations, i.e., x-polarized (along the width W of the nanogap), y-polarized, z-polarized, and a dipole polarized along a direction d oriented at 45° with respect to the x-, y-, and z-axes, are separately considered. These orientations are depicted in Figure S9b. For each dipole orientation at position P_2, the spectrum of ⟨DoCP⟩ on the air side (Figure S9c) and the normalized radiated intensity spectrum on the air side (Figure S9d) are evaluated for an NA of 0.9.

The normalized radiated intensity for the dipolar emitter at P_2 varies with dipole orientation due to differences in coupling efficiency to the nanoantenna modes. The maximal ⟨DoCP⟩ values obtained for each dipole orientation are −0.99 for the x-oriented dipole at 842 nm, −0.97 for the y-oriented dipole at 832 nm, −0.96 for the z-oriented dipole at 848 nm, and −0.99 for the d-oriented dipole at 844 nm. Thus, all four dipole orientations exhibit near-maximal ⟨DoCP⟩ at wavelengths at and near the planar chiral nanoantenna's operating wavelength of 842 nm. At 842 nm, the ⟨DoCP⟩ values are approximately −0.99, −0.96, −0.95, and −0.99 for the x-, y-, z-, and d-oriented dipoles, respectively. The consistently near-maximal ⟨DoCP⟩ at and around 842 nm demonstrates that the emitter-driven planar chiral nanoantenna's far-field response is robust against reasonable variations in both emitter position and orientation within the nanogap, even though the radiated intensity may vary.

VI. Comparative Evaluation of the Proposed Planar Chiral Nanoantenna with Representative Nano-Optical Platforms

Since the main scope of this work pertains to the near-field intensity switchability in the nanogap of the planar chiral nanoantenna and the far-field circularly polarized emission from a single quantum emitter positioned in the nanogap of the same planar chiral nanoantenna, the relevant figures of merit are g, ΔIE, and ⟨DoCP⟩. To estimate the performance of the proposed nanoantenna design, these values have been compared with corresponding values connected to several representative nano-optical platforms.

Aoudjit et al. demonstrate a chiral nanostructure consisting of two shifted gold nanorods separated by a nanogap having very high to near-maximal g at the nanogap.[1] However, it can be inferred from the work that the nanostructures exhibit moderate ΔIE. Zaman et al. show that a plasmonic Archimedean spiral can be employed to switch from a trapping force profile to a rotating force profile by changing the handedness of CPL.[2] This switch is accompanied by a change in the intensity profile at the center of the Archimedean spiral. Even though it is not directly stated, it can be reliably inferred that the near-field intensity switch at the center of the Archimedean spiral is characterized by near-maximal to maximal g but moderate ΔIE. For comparison, the planar chiral nanoantenna proposed in this work not only registers maximal |g| of 2 at 842 nm but also ΔIE of 930.7 at the same wavelength. Gupta et al. take a different route by proposing two different plasmonic achiral nanoantenna geometries situated on top of a thin film made of a phase change material (vanadium dioxide) to achieve strong near-field switching at the nanogap.[3] They report a maximum near-field intensity switching ratio (I_{on}/I_{off}) of 80.85. While this is certainly high, the planar chiral nanoantenna demonstrates a much higher intensity switching ratio ($IE_{R\text{-}CPL}/IE_{L\text{-}CPL}$) of approximately 950.69 at the geometric center of the nanogap at 842 nm.

Ahn et al. demonstrate a single plasmonic helicoid nanoparticle with maximum two-photon-induced photoluminescence |⟨DoCP⟩| of approximately 0.35 (reported |g| ≈ 0.7).[4] Helicity-resolved photoluminescence measurements of multilayer tungsten disulfide spiral nanostructures conducted by Barman et al. suggest near-maximal ⟨DoCP⟩ of approximately 0.94 ± 0.04.[5] The planar chiral nanoantenna reports a maximum |⟨DoCP⟩| of 0.99, thereby matching or beating the reported values associated with comparable chiroptical nanostructures.

Overall, the proposed planar chiral nanoantenna offers a rare combination of maximal dissymmetry in near-field intensity, exceptionally strong absolute contrast in near-field intensity, near-maximal circularly polarized emission, clear design principles, and deterministically fabricable geometry, distinguishing it from representative state-of-the-art nano-optical structures and making it a promising candidate for active chiral nanoplasmonics.

References

[1] T. Aoudjit, A. Horrer, S. Kostcheev, R. Bachelot, J. Plain, D. Gérard, *Adv. Opt. Mater.* **2023**, *11*, 2203015.
[2] M. A. Zaman, P. Padhy, L. Hesselink, *Phys. Rev. A* **2019**, *100*, 013857.
[3] N. Gupta, P. B. Savaliya, A. Dhawan, *Opt. Express* **2020**, *28*, 27476.
[4] H.-Y. Ahn, K. Q. Le, T. Narushima, J. Yamanishi, R. M. Kim, K. T. Nam, H. Okamoto, *Adv. Opt. Mater.* **2024**, *12*, 2400699.
[5] P. K. Barman, P. V. Sarma, M. M. Shaijumon, R. N. Kini, *Sci. Rep.* **2019**, *9*, 2784.
